# Role of Prophylactic Antibiotics in Transperineal Prostate Biopsy: A Systematic Review and Meta-analysis

**DOI:** 10.1016/j.euros.2022.01.001

**Published:** 2022-01-29

**Authors:** Spyridon P. Basourakos, Mark N. Alshak, Patrick J. Lewicki, Emily Cheng, Michael Tzeng, Antonio P. DeRosa, Mathew J. Allaway, Ashley E. Ross, Edward M. Schaeffer, Hiten D. Patel, Jim C. Hu, Michael A. Gorin

**Affiliations:** a Department of Urology, New York Presbyterian Hospital/Weil Cornell Medicine, New York, NY, USA; b Samuel J. Wood Library & C.V. Starr Biomedical Information Center, Weill Cornell Medicine, New York, NY, USA; c Urology Associates and UPMC Western Maryland, Cumberland, MD, USA; d Department of Urology, Northwestern University Feinberg School of Medicine, Chicago, IL, USA; e Department of Urology, Loyola University Medical Center, Maywood, IL, USA; f Department of Urology, University of Pittsburgh School of Medicine, Pittsburgh, PA, USA

**Keywords:** Transperineal, Prostate, Biopsy, Prostate cancer, Antibiotics, Prophylaxis

## Abstract

**Context:**

Transperineal prostate biopsy is associated with a significantly lower risk of infectious complications than the transrectal approach. In fact, the risk of infectious complications with transperineal prostate biopsy is so low that the utility of administering periprocedural antibiotics with this procedure has come under question.

**Objective:**

To perform a systematic review and meta-analysis to assess for differences in the rates of infectious complications (septic, nonseptic, and overall) after performing transperineal prostate biopsy with and without the administration of periprocedural antibiotic prophylaxis.

**Evidence acquisition:**

Three electronic databases (PubMed, Embase, and MEDLINE) were searched, and studies were included if they included patients who underwent transperineal prostate biopsy, were published after January 2000, included information on periprocedural antibiotic administration, and reported postbiopsy complications. Preferred Reporting Items for Systematic Reviews and Meta-analyses and Agency for Healthcare Research and Quality guidelines were utilized.

**Evidence synthesis:**

A total of 106 unique studies describing 112 cohorts of patients were identified, of which 98 (37 805 men) received antibiotic prophylaxis and 14 (4772 men) did not receive it. All patients were included in the analysis of septic complications. In total, there were 19/37 805 (0.05%) episodes of sepsis in the group of men who received antibiotics, which was similar to the no antibiotic group with 4/4772 (0.08%) episodes (*p* = 0.2). For overall infections (septic plus nonseptic), there were 403/29 880 (1.35%) versus 58/4772 (1.22%) events among men with evaluable data who received and did not receive antibiotic prophylaxis, respectively (*p* = 0.8). Restricting our analysis to studies with a comparable low number of biopsy cores (<25 cores), there remained no difference in the rates of sepsis between groups, but there was a small, statistically significant lower risk of infectious complications with antibiotic administration—67/12 140 (0.55%) versus 58/4772 (1.22%; *p* < 0.01).

**Conclusions:**

The likelihood of septic infections after transperineal prostate biopsy is low with and without antibiotic prophylaxis. The omission of periprocedural antibiotics with this procedure stands to benefit patients by avoiding potential drug reactions. Furthermore, this practice is in line with calls throughout the medical community for improved antibiotic stewardship.

**Patient summary:**

In a large systematic review and meta-analysis, we evaluated infectious complications after transperineal prostate biopsy with or without the administration of prophylactic antibiotics. We conclude that prophylactic antibiotics do not decrease the rate of postbiopsy sepsis but may have a small benefit in terms of preventing less serious infections.

## Take Home Message

The likelihood of septic infections after transperineal prostate biopsy is similarly low with and without antibiotic prophylaxis. The omission of periprocedural antibiotics with this procedure stands to benefit patients by avoiding potential drug reactions associated with their use.

## 1 Introduction

Transrectal prostate biopsy is the current mainstay of prostate cancer diagnosis in most areas of the world [1], [2]. Despite the use of targeted and/or augmented antibiotic regimens for periprocedural prophylaxis, transrectal prostate biopsy is associated with a significant risk of infectious complications, with the overall incidence in the range of 5–7% [3]. In contrast, transperineal prostate biopsy, which is performed percutaneously, thereby avoiding contact of the biopsy needle with the rectal mucosa, carries a risk of infectious complications that is approximately half of that of the transrectal approach. An analysis of the combined data from seven randomized trials comparing the two biopsy approaches with respect to overall infectious complications reported a risk ratio of 0.55 (95% confidence interval 0.33–0.92), favoring transperineal prostate biopsy [4]. When examining the risk of sepsis in particular, one meta-analysis, which included approximately 160 000 patients, placed the incidence of this complication at only 0.1% with the transperineal approach [5]. This figure was eight times higher (0.8%) than that with transrectal prostate biopsy.

As a result of the mounting data favoring transperineal prostate biopsy, recently the European Association of Urology (EAU) released a position paper as well as guideline recommendations that endorsed the use of this procedure whenever technically feasible in place of the transrectal approach [6], [7]. This recommendation has been welcomed with open arms by the “TRexit” movement, which advocates for complete abandonment of transrectal prostate biopsy [8], [9]. In fact, some have gone so far as to state that the safety profile of transperineal prostate biopsy warrants its widespread adoption without the use antibiotic prophylaxis. Proponents of this cite improved antibiotic stewardship as well as the elimination of antibiotic-related adverse events as the rationale for their view. Indeed, there is evidence of safely foregoing prophylactic antibiotics with transperineal prostate biopsy [10], [11], [12], [13], [14]. It is worth acknowledging, however, that much of the available data come from single-arm cohort studies without a comparison with the use of periprocedural antibiotics. With these questions in mind, we set out to perform a systematic review and meta-analysis with the primary aim of comparing the rates of infectious complications with these two competing practices.

## 2 Evidence acquisition

### 2.1 Search strategy

This study was registered with PROSPERO, the international prospective registrar of systematic reviews (registration number: CRD42021228477), and followed the guidelines set forth by the Preferred Reporting Items for Systematic Reviews and Meta-analyses (PRISMA) statement and Agency for Healthcare Research and Quality (AHRQ) Methods Guide for Effectiveness and Comparative Effectiveness Reviews [15], [16].

Comprehensive literature searches were conducted on December 29, 2020, in three databases for any publication types and reports of human studies after January 2000. The databases searched were MEDLINE (via PubMed), Embase (via OVID), and the Cochrane Library (via Wiley). Controlled vocabularies and text words were used in the development of the search strategies in all databases. Search results were combined in a bibliographic management tool (EndNote), and duplicates were removed both electronically and through a manual review. Our initial database search produced 1628 results, which were imported in Covidence (Veritas Health Innovation, Melbourne, Australia), a systematic review support tool, for further management and review, which included title/abstract screening and full-text screening phases. A focused update and manual review identified two additional articles for inclusion through April 30, 2021 [13], [17].

The search terminology included four major concepts, all linked together with the AND operator: (1) transperineal, perineal, or perineum; (2) biopsy or biopsies including large and fine needle; (3) prostate cancer or prostatic neoplasia; and (4) infections including fever, sepsis, abscess, urinary tract infection (UTI), prostatitis, and others. Septic infections were identified as reported by authors of each study. To incorporate the gray literature perspective, publication types from Embase such as conference proceedings, technical and other reports, and theses/dissertations were screened. For a complete list of medical subject headings (MeSH) and keyword terms used in search strategy development, please refer to the MEDLINE search strategy accompanying this paper (Supplementary material).

### 2.2 Study criteria

**Fig. 1:**
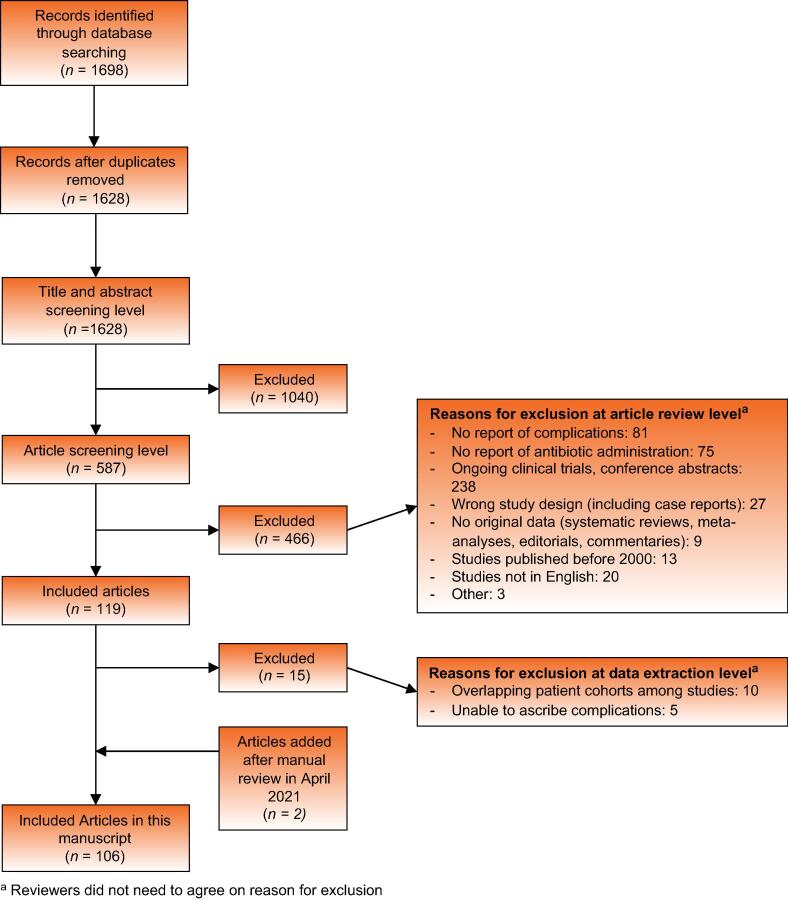
Summary of the literature search. ^a^Reviewers did not need to agree on reason for exclusion.

A total of 1698 citations were screened by title and abstract against predetermined inclusion and exclusion criteria by two independent reviewers. Any discrepancies were resolved by consensus. A total of 587 articles were selected for full-text review, and 106 of these articles met the inclusion criteria for this study. Figure 1 outlines the study selection process. A careful exclusion of duplicates by institution and year was performed as feasible. Articles with overlapping cohorts were evaluated separately by two reviewers, and we included only the most comprehensive publications.

### 2.3 Extracted variables and endpoint

Three independent investigators extracted data from all the selected studies. A standardized data extraction sheet defined a priori by the study team was utilized. Extracted data, where applicable, included study type (prospective vs retrospective and cohort vs randomized controlled trial [RCT]), country of senior author, number of participants, median/mean age, type of transperineal biopsy (ultrasound guided vs ultrasound/magnetic resonance imaging fusion), average number of biopsy cores, type of anesthesia (general, local, sedation, spinal, or combination), use of periprocedural antibiotics, duration and scheme of antibiotic prophylaxis if used, events of sepsis, and events of other infectious complications (fever, UTI, prostatitis, epididymo-orchitis, pyelonephritis, and unspecified infection). The primary outcomes of interest were septic and overall (septic plus nonseptic) infectious complications within 30 d after biopsy.

### 2.4 Statistical analysis

The two study groups of interest were patients who underwent transperineal prostate biopsy (1) with and (2) without prophylactic periprocedural antibiotics. Measures of central tendency (mean or median) as reported in individual studies were summarized as the median and interquartile range (IQR) across included studies for the variables of age and number of biopsy cores. The total proportions of postbiopsy infections (septic, nonseptic, and overall) were tabulated for each group with pooled samples compared by two-sample tests of proportions. One-sided *p* values of <0.05 were considered statistically significant to assess whether infectious complications were higher in the group that did not receive prophylactic antibiotics.

A meta-analysis was conducted for the outcomes of septic, nonseptic, and overall infectious complications after biopsy using random-effect modeling. Freeman-Tukey double arcsine transformation was applied to stabilize variances for binomial data [18]. A subgroup analysis was conducted by stratifying the number of biopsy cores. Analyses were conducted using STATA version 15.0 (2017; STATA Corp, College Station, TX, USA).

### 2.5 Risk of bias and strength of evidence assessment

Risk of bias was assessed based on AHRQ guidelines, which suggest evaluation of design, enrollment/exposure, and outcome assessment for noncomparative single-arm studies [16], [18]. Three investigators independently rated the included studies considering three items: design (specifying details on prostate biopsy template and antibiotic use), consecutive enrollment, and objective measurement of outcome (sufficient follow-up and method of assessment). If all three items were rated favorably, the study was considered to be of high quality. If one item was unfavorable or unclear, the study was considered to be of moderate quality. If two or all three items were unfavorable or unclear, the study was considered to be of low quality. We graded the strength of evidence using the AHRQ EPC Methods Guide for Conducting Effectiveness and Comparative Effectiveness Reviews scheme [16].

## 3 Evidence synthesis

### 3.1 Study design

From 1628 citations screened, we identified a total of 106 unique studies describing 112 cohorts of patients eligible for a quantitative analysis. Six (5.7%) studies described mixed cohorts of patients contributing to both study groups. There were 47 (44.3%) retrospective cohort studies, 45 prospective (42.5%) studies, and 14 (13.2%) RCTs. No randomized trials were focused on comparing antibiotic prophylaxis with no antibiotic prophylaxis for transperineal prostate biopsy. Twelve studies (11.3%) were multicenter. In total, 37 805 men from 98 patient cohorts received antibiotic prophylaxis. An additional 4772 men from 14 cohorts did not receive antibiotic prophylaxis (Fig. 1, Table 1, and Supplementary Table 1).

**Table 1:** Summary of data for studies with men undergoing transperineal prostate biopsy without prophylactic antibiotics

Study name (year)	Country	Sample size (*n*)	Type of study	Age (yr)	Mean or median number of biopsy cores (*n*)	Anesthesia	Sepsis (*n*)	Nonseptic infections (*n*)	All infections (*n*)
Meyer et al (2018) [66]	USA	43	Retrospective cohort	Median 62 (range 44–73)	12.6	Local only	0	0	0
Ristau et al (2018) [87]^a^	USA	400	Retrospective cohort	Median 68 (IQR 61–74)	16	Local and sedation	0	0	0
Gorin et al (2020) [11]	USA	94	Prospective cohort	Median 68.8 (range 52–86.4)	12	Local only or sedation only	0	0	0
Wetterauer et al (2020) [113]^a^	Switzerland	177	Retrospective cohort	Median 66 (range 49–86)	13	Local only	0	0	0
Szabo (2021) [100]^a^	USA	212	Retrospective cohort	Median 63 (range 29–93)	20	Local only	0	0	0
John et al (2021) [13]^a^	UK	164	Prospective cohort	Median 71 (IQR 67–75)	23	Local only	0	0	0
Lopez et al (2021) [14]^a^	UK, New Zealand, Hong Kong	175	Prospective cohort	Median 68 (IQR 62–72)	24	Local only	0	0	0
Miller et al (2005) [69]	Australia	81	Retrospective cohort	Mean 69.5 (95% CI 68.1–70.9)	18	Local only	1	0	1
Dimmen et al (2012) [10]	Norway	69	Retrospective cohort	Median 64.5 (range 50–78)	18.4	Local and sedation	1	1	2
Jacewicz et al (2020) [12]^a^	Multinational	230	Retrospective cohort	Mean 67 (95% CI 66–68)	NR	Local only	1	1	2
Sigle et al (2021) [91]	Germany	184	Retrospective cohort	Median 66.9 (IQR 61.8–72.0)	41	General	0	2	2
Gunzel et al (2021) [17]	Germany	621	Retrospective cohort	Median 68 (IQR 62–74)	10	Local only	1	3	4
Huang et al (2019) [46]	Taiwan	130	RCT	Mean 66.6 (SD 8.81)	10	General or local only	0	6	6
Ding et al (2021) [34]	China	2192	Retrospective cohort	Mean 67.63 (SD 7.11)	22	NR	0	41	41

CI = confidence interval; IQR = interquartile range; NR = not recorded; RCT = randomized controlled trial; SD = standard deviation.

a Studies with cohorts of men undergoing transperineal prostate biopsy with and without prophylactic antibiotics.

### 3.2 Studies reporting transperineal prostate biopsies with the use of antibiotics

We found 39 retrospective studies, 46 prospective studies, and 13 RCTs where periprocedural antibiotics were administered [12], [13], [14], [19], [20], [21], [22], [23], [24], [25], [26], [27], [28], [29], [30], [31], [32], [33], [35], [36], [37], [38], [39], [40], [41], [42], [43], [44], [45], [47], [48], [49], [50], [51], [52], [53], [54], [55], [56], [57], [58], [59], [60], [61], [62], [63], [64], [65], [67], [68], [70], [71], [72], [73], [74], [75], [76], [77], [78], [79], [80], [81], [82], [83], [84], [85], [86], [87], [88], [89], [90], [92], [93], [94], [95], [96], [97], [98], [99], [100], [101], [102], [103], [104], [105], [106], [107], [108], [109], [110], [111], [112], [113], [114], [115], [116], [117], [118]. The study sizes range from 16 to 3007 men. In 53 (54.1%) studies, fluoroquinolones were the antibiotics of choice, either alone or in combination with another antibiotic. The second most used antibiotic were aminoglycosides, which were used in 16 (16.3%) of the studies. The range of antibiotic coverage was from the day before biopsy to 7 d after the biopsy. The median patient age across studies was 66 (IQR: 63.8–68) yr, and the median number of biopsy cores taken was 24 (IQR: 16–32).

All 37 805 patients contributed to the outcome of sepsis, with 19 (0.05%) experiencing an event (Supplementary Table 1). A total of 29 880 men contributed to the outcomes of nonseptic and overall infections, with 388 (1.13%) and 403 (1.35%) events for each of these outcomes, respectively.

### 3.3 Studies reporting transperineal prostate biopsies without the use of antibiotics

We found ten retrospective studies, three prospective studies, and one RCT where antibiotic prophylaxis was not administered [10], [11], [12], [13], [14], [17], [34], [46], [66], [69], [87], [91], [100], [113]. The study cohorts included 43–2192 men. Five (36%) of these studies were performed in Europe, four (29%) in the USA, two (14%) in Asia, and one (7%) in Australia, and two (14%) were multinational studies. Across eight studies, 1703 (36%) biopsies were performed only under local anesthesia. The median patient age across studies was 67.3 (IQR: 66–68) yr, and the median number of biopsy cores taken was 18 (IQR: 12.6–22). All 4772 men contributed to the outcomes of sepsis, nonseptic infection, and overall infections, with a total of four (0.08%), 54 (1.13%), and 58 (1.22%) events for each of these outcomes, respectively (Table 1).

### 3.4 Pooled comparison and meta-analysis

**Fig. 2:**
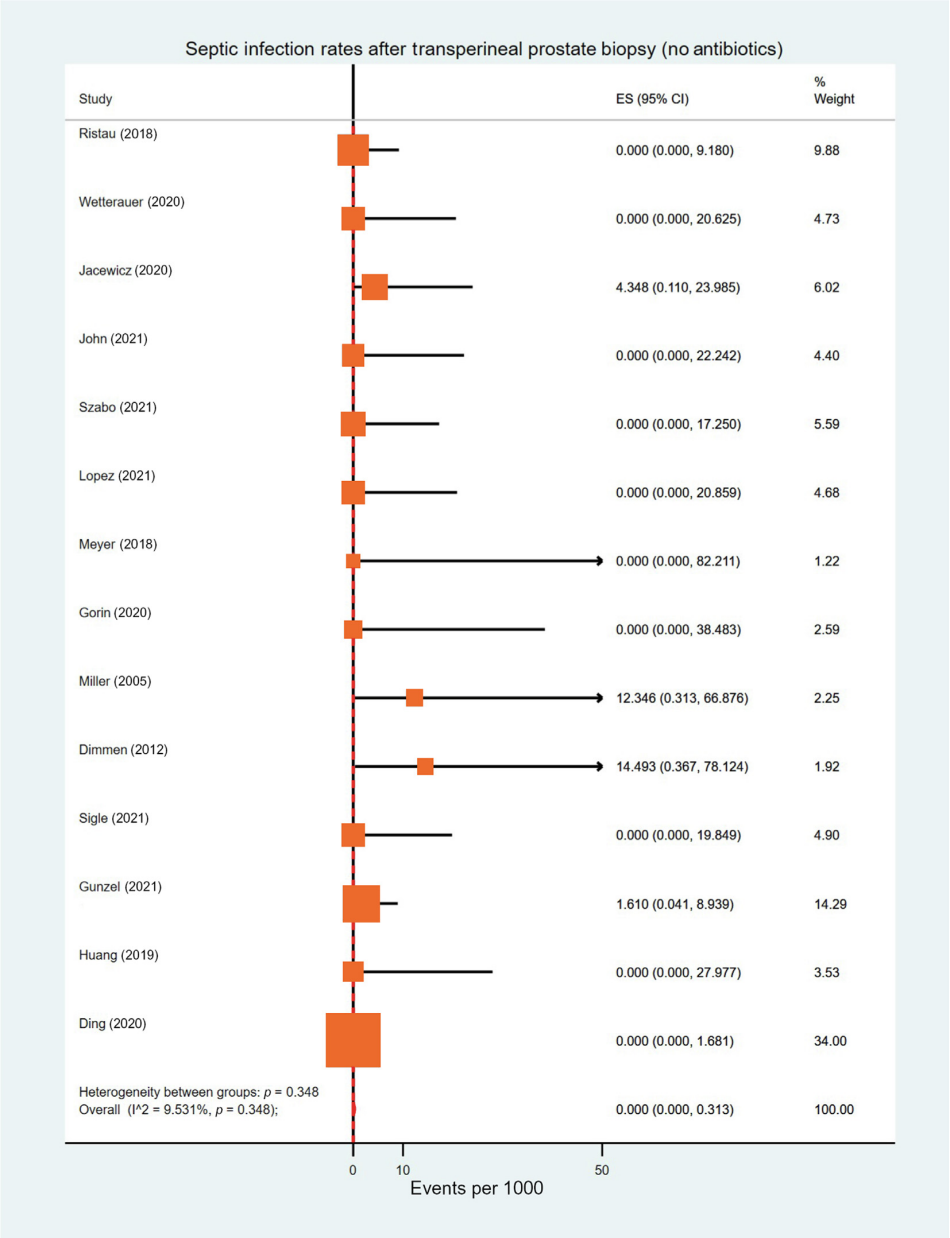
A meta-analysis for the proportion of men who underwent transperineal prostate biopsy without receiving periprocedural prophylactic antibiotics and developed postprocedural sepsis (I^2^ = 9.5%, *p* = 0.348). CI = confidence interval; ES = effect size.

Overall, two-sample tests of proportions did not demonstrate the rate of septic infections to be significantly higher in the groups with and without antibiotic prophylaxis (0.05% vs 0.08%, *p* = 0.2). Additionally, the rate of overall infections was similar between the two groups (1.35% vs 1.22%, *p* = 0.8). A meta-analysis of proportions across studies led to negligible effect sizes for sepsis events in either group (0.00 per 1000; Fig. 2 and Supplementary Figure 3). For men who received antibiotic prophylaxis, meta-analysis effect sizes for nonseptic and overall infections were 5.65 and 6.32 per 1000, respectively. In the group that did not receive antibiotics, these figures were 3.26 and 4.66 per 1000, respectively (Supplementary Fig. 1, 2, 4, and 5).

### 3.5 Subgroup analysis by number of biopsy cores

Given the higher number of biopsy cores taken among studies using antibiotic prophylaxis (median of 24 vs 18, *p* = 0.01), studies were stratified by the number of biopsy cores. We identified 42 (39.6%) studies reporting a mean or median of <25 cores (“low” group; median 18 [IQR: 14–21.2]) and 33 studies with ≥25 cores (“high” group; median 36 [IQR: 30–54]). The rate of sepsis was similar between the low and high groups (0.07% vs 0.09%, *p* = 0.3), but a high number of biopsy cores was associated with an increased risk of overall infections (2.64% vs 0.55%, *p* < 0.01). When comparing the antibiotic and no antibiotic groups within the low biopsy core stratum, rates of sepsis were comparable (12/16 081 [0.07%] vs 4/4772 [0.08%], *p* = 0.4), whereas the rate of overall infections was higher in the no prophylaxis group (67/12 140 [0.55%] vs 58/4772 [1.22%], *p* < 0.01; Table 1).

### 3.6 Risk of bias assessment

**Fig. 3:**
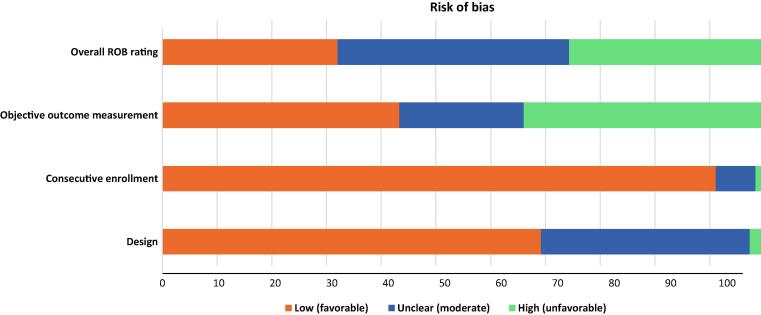
Risk of bias assessment and strength of evidence. ROB = risk of bias.

Across the 106 unique studies that were evaluated, 31 (29.2%), 41 (38.7%), and 34 (32.1%) were identified as having a low, moderate, and high risk of bias, respectively (Fig. 3). The strength of evidence was rated to be moderate due to medium study limitations, direct outcome measurement, consistent but imprecise event rates, and an undetected reporting bias.

### 3.7 Discussion

In this meta-analysis, which included over 42 000 men across 106 unique studies, we found no significant differences in septic, nonseptic, and overall infectious complications after transperineal prostate biopsy in the presence or absence of periprocedural antibiotic prophylaxis. This trend held with respect to sepsis and after accounting for difference in biopsy core numbers between groups; however, we observed a higher risk of overall infections in the no antibiotic prophylaxis group. Although statistically significant, the absolute difference in terms of overall infectious complications was <1%, which may be considered clinically insignificant by most clinicians considering that sepsis rates were maintained at <0.1%.

The similar rates of sepsis with and without antibiotics identified in this review suggest that antibiotics may have minimal impact on the prevention of serious infectious complications after transperineal biopsy. In light of this finding, we feel that consideration should be given to performing this biopsy procedure without antibiotic prophylaxis. This potentially stands to benefit antibiotic stewardship, as bacterial resistance to fluoroquinolones, the most commonly used antibiotic for transrectal biopsy prophylaxis, has risen steadily in recent decades [3]. More specifically, Cohen et al [119] reported that one out of every four men on prostate cancer active surveillance now harbors rectal flora resistant to fluoroquinolones. By eliminating the use of prophylaxis for transperineal prostate biopsy, less selective pressure should be placed on bacteria to develop a mechanism for antibiotic resistance.

The exclusion of antibiotics when they are not beneficial is also cost effective. While the cost of commonly used antibiotics for prostate biopsy prophylaxis as well as rectal swabs is relatively low, considering the number of prostate biopsies performed annually, it becomes a significant burden for healthcare systems [120], [121]. Additionally, the omission of unnecessary antibiotics for patients undergoing a transperineal prostate biopsy can spare patients from potential adverse drug reactions such as nephrotoxicity from aminoglycosides and musculoskeletal complications from fluoroquinolones [122], [123]. Transitioning from general to local anesthesia for transperineal biopsy will also impact costs, although we found that only 36% of cases were currently performed with local anesthesia in the no antibiotics groups.

There has been considerable discussion in the literature about the number of biopsy cores taken and its association with infectious complications. When the transrectal approach is performed, the number of cores has no significant relationship with postbiopsy infection rates [5], [124], [125]. However, for the transperineal approach, we showed that the number of cores directly correlated with infection rates, such that there were more nonseptic infection events in the high biopsy core strata. Nevertheless, the rate of sepsis remained <0.1% and was not affected by the quantity of core samples obtained. It is possible that prior studies were limited by sample size and low event rates.

### 3.8 Summary of key findings

There is a growing interest in the use of transperineal prostate biopsy among the urological community. Unlike with the transrectal approach, it is unclear whether the use of periprocedural prophylactic antibiotics is warranted when performing transperineal prostate biopsy. Our meta-analysis of 106 different studies indicated that the rate of infectious complications for transperineal biopsy with and without antibiotics is around 1 with a <0.1% risk of sepsis. No statistically significant difference was found in the rate of septic infections between the two cohorts, indicating that there is likely limited benefit to providing patients with periprocedural antibiotics.

### 3.9 Limitations

This study is not without limitations. Most noteworthy is the fact that the majority of studies included in our analysis were retrospective and/or single arm in design, without any direct comparison between the two groups of interest. A second related limitation is the fact that studies of mixed methodological design were included in the analysis. This may have introduced a bias, as it is likely that data derived from RCTs and prospective studies had more intensive and reliable follow-up than data derived from retrospective reports. Given the greater proportion of patient data derived from studies of lower methodological quality in the no antibiotics group, it is certainly possible that the equivalence in outcomes was an artifact of this bias. It is reassuring, however, that the rate of infectious complications was overall low across studies regardless of methodological design, and therefore it is unlikely that this factor confounded our analysis. One final limitation is that our analysis did not take into account potential differences in patient-level data such as medical comorbidities, prior exposure to a transrectal biopsy, or number of prior prostate biopsies, all factors that can contribute to postprocedural infections [126], [127]. For sepsis events, the susceptibility profile of the offending organism was not reported consistently. Again, the overall number of events in this study was low, and so it is unlikely that the knowledge of these factors would have impacted the results significantly.

## 4 Conclusions

In a meta-analysis with data from over 42 000 patients comparing the rate of infectious complications following transperineal prostate biopsy with and without periprocedural prophylactic antibiotics, we found no statistically or clinically significant differences in the rates of sepsis or overall infections between groups. In the low (<25) biopsy core stratum, there remained no significant difference in the rate of sepsis and a <1% absolute risk reduction for overall infections with prophylactic antibiotics. Given the low rate of infections, omission of periprocedural antibiotics stands to benefit antibiotic stewardship and avoidance of potential drug reactions. Clinical trials are deemed necessary to further validate our findings.

  ***Author contributions*:** Spyridon P. Basourakos had full access to all the data in the study and takes responsibility for the integrity of the data and the accuracy of the data analysis.

*Study concept and design*: Basourakos, Alshak, Lewicki, DeRosa, Hu, Gorin.

*Acquisition of data*: Basourakos, Alshak, Lewicki, Cheng, Tzeng, DeRosa.

*Analysis and interpretation of data*: Basourakos, Alshak, Lewicki, Cheng, Tzeng, Allaway, Ross, Schaeffer, Patel, Hu, Gorin.

*Drafting of the manuscript*: Basourakos, Cheng, Tzeng, Patel, Gorin.

*Critical revision of the manuscript for important intellectual content*: Basourakos, Allaway, Ross, Schaeffer, Patel, Hu, Gorin.

*Statistical analysis*: Patel.

*Obtaining funding*: Hu, Gorin.

*Administrative, technical, or material support*: Hu, Gorin.

*Supervision*: Hu, Gorin.

*Other*: None.

  ***Financial disclosures:*** Spyridon P. Basourakos certifies that all conflicts of interest, including specific financial interests and relationships and affiliations relevant to the subject matter or materials discussed in the manuscript (eg, employment/affiliation, grants or funding, consultancies, honoraria, stock ownership or options, expert testimony, royalties, or patents filed, received, or pending), are the following: Mathew J. Allaway is the founder and co-owner of Perineologic. Michael A. Gorin is a paid consultant to BK Medical ApS, KOELIS, Inc., and Perineologic.

  ***Funding/Support and role of the sponsor*:** Jim C. Hu receives research support from the Frederick J. and Theresa Dow Wallace Fund of the New York Community Trust. Jim C. Hu also receives salary support from NIH R01 CA241758, PCORI CER-2019C1-15682, and CER-2019C2-17372. The remaining authors report no further disclosures related to this work.

## References

[1] Johansen T.E.B., Zahl P.H., Baco E. (2020). Antibiotic resistance, hospitalizations, and mortality related to prostate biopsy: first report from the Norwegian Patient Registry. World J Urol.

[2] Borghesi M., Ahmed H., Nam R. (2017). Complications after systematic, random, and image-guided prostate biopsy. Eur Urol.

[3] Liss M.A., Ehdaie B., Loeb S. (2017). An update of the American Urological Association white paper on the prevention and treatment of the more common complications related to prostate biopsy. J Urol.

[4] Pradere B., Veeratterapillay R., Dimitropoulos K. (2021). Nonantibiotic strategies for the prevention of infectious complications following prostate biopsy: a systematic review and meta-analysis. J Urol.

[5] Bennett H.Y., Roberts M.J., Doi S.A., Gardiner R.A. (2016). The global burden of major infectious complications following prostate biopsy. Epidemiol Infect.

[6] Pilatz A., Veeratterapillay R., Dimitropoulos K. (2021). European Association of Urology position paper on the prevention of infectious complications following prostate biopsy. Eur Urol.

[7] Mottet N., van den Bergh R.C.N., Briers E. (2021). EAU-EANM-ESTRO-ESUR-SIOG guidelines on prostate cancer—2020 update. Part 1: screening, diagnosis, and local treatment with curative intent. Eur Urol.

[8] Grummet J., Gorin M.A., Popert R. (2020). “TREXIT 2020”: why the time to abandon transrectal prostate biopsy starts now. Prostate Cancer Prostatic Dis.

[9] Grummet J.P., Mottet N., Gorin M.A. (2021). TREXIT is now: should we abandon the transrectal route for prostate biopsy?. Yes. Eur Urol Open Sci.

[10] Dimmen M., Vlatkovic L., Hole K.H., Nesland J.M., Brennhovd B., Axcrona K. (2012). Transperineal prostate biopsy detects significant cancer in patients with elevated prostate-specific antigen (PSA) levels and previous negative transrectal biopsies. BJU Int.

[11] Gorin M.A., Meyer A.R., Zimmerman M. (2020). Transperineal prostate biopsy with cognitive magnetic resonance imaging/biplanar ultrasound fusion: description of technique and early results. World J Urol.

[12] Jacewicz M., Gunzel K., Rud E. (2021). Multicenter transperineal MRI-TRUS fusion guided outpatient clinic prostate biopsies under local anesthesia. Urol Oncol.

[13] John JB, MacCormick A, MacDonagh R, Speakman MJ, Vennam R, Burns-Cox N. Complications following local anaesthetic transperineal prostate biopsies without antibiotic prophylaxis: an institution’s experience. J Clin Urol. In press. 10.1177/2051415820987661.

[14] Lopez J.F., Campbell A., Omer A. (2021). Local anaesthetic transperineal (LATP) prostate biopsy using a probe-mounted transperineal access system: a multicentre prospective outcome analysis. BJU Int.

[15] Liberati A., Altman D.G., Tetzlaff J. (2009). The PRISMA statement for reporting systematic reviews and meta-analyses of studies that evaluate healthcare interventions: explanation and elaboration. BMJ.

[16] AHRQ. Methods guide for effectiveness and comparative effectiveness reviews. AHRQ Publication No. 10(14)-EHC063-EF. Rockville, MD: Agency for Healthcare Research and Quality; 2014. 21433403

[17] Gunzel K., Magheli A., Baco E. (2021). Infection rate and complications after 621 transperineal MRI-TRUS fusion biopsies in local anesthesia without standard antibiotic prophylaxis. World J Urol.

[18] Patel H.D., Gupta M., Cheaib J.G. (2020). Testis-sparing surgery and scrotal violation for testicular masses suspicious for malignancy: a systematic review and meta-analysis. Urol Oncol.

[19] Asano T., Kobayashi S., Yano M., Otsuka Y., Kitahara S. (2015). Continued administration of antithrombotic agents during transperineal prostate biopsy. Int Braz J Urol.

[20] Baba K., Sekine Y., Miyazawa Y. (2018). Assessment of antimicrobial prophylaxis in transperineal prostate biopsy: a single-center retrospective study of 485 cases. J Infect Chemother.

[21] Babaei Jandaghi A., Habibzadeh H., Falahatkar S., Heidarzadeh A., Pourghorban R. (2016). Transperineal prostate core needle biopsy: a comparison of coaxial versus noncoaxial method in a randomised trial. Cardiovasc Intervent Radiol.

[22] Bass E.J., Donaldson I.A., Freeman A. (2017). Magnetic resonance imaging targeted transperineal prostate biopsy: a local anaesthetic approach. Prostate Cancer Prostatic Dis.

[23] Bhatt N.R., Breen K., Haroon U.M., Akram M., Flood H.D., Giri S.K. (2018). Patient experience after transperineal template prostate biopsy compared to prior transrectal ultrasound guided prostate biopsy. Cent European J Urol.

[24] Bigliocchi M., Marini M., Nofroni I., Perugia G., Shahabadi H., Ciccariello M. (2007). Prostate cancer detection rate of transrectal ultrasonography, digital rectal examination, and prostate-specific antigen: results of a five-year study of 6- versus 12-core transperineal prostate biopsy. Minerva Urol Nefrol.

[25] Bittner N., Merrick G.S., Bennett A. (2015). Diagnostic performance of initial transperineal template-guided mapping biopsy of the prostate gland. Am J Clin Oncol.

[26] Bittner N., Merrick G.S., Butler W.M., Bennett A., Galbreath R.W. (2013). Incidence and pathological features of prostate cancer detected on transperineal template guided mapping biopsy after negative transrectal ultrasound guided biopsy. J Urol.

[27] Bott S.R., Henderson A., Halls J.E., Montgomery B.S., Laing R., Langley S.E. (2006). Extensive transperineal template biopsies of prostate: modified technique and results. Urology.

[28] Cerruto M.A., Vianello F., D'Elia C., Artibani W., Novella G. (2014). Transrectal versus transperineal 14-core prostate biopsy in detection of prostate cancer: a comparative evaluation at the same institution. Arch Ital Urol Androl.

[29] Chiu P.K., Lo K.L., Teoh J.Y. (2021). Sectoral cancer detection and tolerability of freehand transperineal prostate biopsy under local anaesthesia. Prostate Cancer Prostatic Dis.

[30] Cronin T., Neill L., Nelson J., Stewart R., Sangster P., Khoubehi B. (2017). Complications of transperineal template-guided prostate biopsy: a single centre experience in 109 cases. Surg Pract.

[31] Danforth T.L., Chevli K.K., Baumann L., Duff M. (2012). Low incidence of prostate cancer identified in the transition and anterior zones with transperineal biopsy. Res Rep Urol.

[32] Demura T., Hioka T., Furuno T. (2005). Differences in tumor core distribution between palpable and nonpalpable prostate tumors in patients diagnosed using extensive transperineal ultrasound-guided template prostate biopsy. Cancer.

[33] DiBianco J.M., Mullins J.K., Allaway M. (2016). Ultrasound guided, freehand transperineal prostate biopsy: an alternative to the transrectal approach. Urol Pract.

[34] Ding X.F., Luan Y., Lu S.M. (2021). Risk factors for infection complications after transrectal ultrasound-guided transperineal prostate biopsy. World J Urol.

[35] Ekwueme K., Simpson H., Zakhour H., Parr N.J. (2013). Transperineal template-guided saturation biopsy using a modified technique: outcome of 270 cases requiring repeat prostate biopsy. BJU Int.

[36] Eldred-Evans D., Kasivisvanathan V., Khan F. (2016). The use of transperineal sector biopsy as a first-line biopsy strategy: a multi-institutional analysis of clinical outcomes and complications. Urol J.

[37] Emiliozzi P., Longhi S., Scarpone P., Pansadoro A., DePaula F., Pansadoro V. (2001). The value of a single biopsy with 12 transperineal cores for detecting prostate cancer in patients with elevated prostate specific antigen. J Urol.

[38] Emiliozzi P., Scarpone P., DePaula F. (2004). The incidence of prostate cancer in men with prostate specific antigen greater than 4.0 ng/ml: a randomized study of 6 versus 12 core transperineal prostate biopsy. J Urol.

[39] Furuno T., Demura T., Kaneta T. (2004). Difference of cancer core distribution between first and repeat biopsy: In patients diagnosed by extensive transperineal ultrasound guided template prostate biopsy. Prostate.

[40] Garcia Bennett J., Vilanova J.C., Guma Padro J., Parada D., Conejero A. (2017). Evaluation of MR imaging-targeted biopsies of the prostate in biopsy-naive patients. A single centre study. Diagn Interv Imaging.

[41] Gershman B., Zietman A.L., Feldman A.S., McDougal W.S. (2013). Transperineal template-guided prostate biopsy for patients with persistently elevated PSA and multiple prior negative biopsies. Urol Oncol.

[42] Guo G., Xu Y., Zhang X. (2017). TRUS-guided transperineal prostate 12+X core biopsy with template for the diagnosis of prostate cancer. Oncol Lett.

[43] Guo L.H., Wu R., Xu H.X. (2015). Comparison between ultrasound guided transperineal and transrectal prostate biopsy: a prospective, randomized, and controlled trial. Sci Rep.

[44] Hadaschik B.A., Kuru T.H., Tulea C. (2011). A novel stereotactic prostate biopsy system integrating pre-interventional magnetic resonance imaging and live ultrasound fusion. J Urol.

[45] Hara R., Jo Y., Fujii T. (2008). Optimal approach for prostate cancer detection as initial biopsy: prospective randomized study comparing transperineal versus transrectal systematic 12-core biopsy. Urology.

[46] Huang G.L., Kang C.H., Lee W.C., Chiang P.H. (2019). Comparisons of cancer detection rate and complications between transrectal and transperineal prostate biopsy approaches—a single center preliminary study. BMC Urol.

[47] Huang H., Wang W., Lin T. (2016). Comparison of the complications of traditional 12 cores transrectal prostate biopsy with image fusion guided transperineal prostate biopsy. BMC Urol.

[48] Huang S., Reeves F., Preece J., Satasivam P., Royce P., Grummet J.P. (2015). Significant impact of transperineal template biopsy of the prostate at a single tertiary institution. Urol Ann.

[49] Igel T.C., Knight M.K., Young P.R. (2001). Systematic transperineal ultrasound guided template biopsy of the prostate in patients at high risk. J Urol.

[50] Iremashvili V.V., Chepurov A.K., Kobaladze K.M., Gamidov S.I. (2010). Periprostatic local anesthesia with pudendal block for transperineal ultrasound-guided prostate biopsy: a randomized trial. Urology.

[51] Klatte T., Swietek N., Schatzl G., Waldert M. (2013). Transperineal template-guided biopsy for diagnosis of prostate cancer in patients with at least two prior negative biopsies. Wien Klin Wochenschr.

[52] Kum F., Elhage O., Maliyil J. (2020). Initial outcomes of local anaesthetic freehand transperineal prostate biopsies in the outpatient setting. BJU Int.

[53] Kum F., Jones A., Nigam R. (2019). Factors influencing urinary retention after transperineal template biopsy of the prostate: outcomes from a regional cancer centre. World J Urol.

[54] Kuru T.H., Roethke M.C., Seidenader J. (2013). Critical evaluation of magnetic resonance imaging targeted, transrectal ultrasound guided transperineal fusion biopsy for detection of prostate cancer. J Urol.

[55] Li H., Yan W., Zhou Y., Ji Z., Chen J. (2007). Transperineal ultrasound-guided saturation biopsies using 11-region template of prostate: report of 303 cases. Urology.

[56] Lo K.L., Chui K.L., Leung C.H. (2019). Outcomes of transperineal and transrectal ultrasound-guided prostate biopsy. Hong Kong Med J.

[57] Losa A., Gadda G.M., Lazzeri M. (2013). Complications and quality of life after template-assisted transperineal prostate biopsy in patients eligible for focal therapy. Urology.

[58] Mabjeesh N.J., Lidawi G., Chen J., German L., Matzkin H. (2012). High detection rate of significant prostate tumours in anterior zones using transperineal ultrasound-guided template saturation biopsy. BJU Int.

[59] Mai Z., Yan W., Zhou Y. (2016). Transperineal template-guided prostate biopsy: 10 years of experience. BJU Int.

[60] Marra G., Zhuang J., Beltrami M. (2021). Transperineal freehand multiparametric MRI fusion targeted biopsies under local anaesthesia for prostate cancer diagnosis: a multicentre prospective study of 1014 cases. BJU Int.

[61] Martorana E., Micali S., Ghaith A. (2015). Advantages of single-puncture transperineal saturation biopsy of prostate: analysis of outcomes in 125 patients using our scheme. Int Urol Nephrol.

[62] Mehmood K., Mubarak M., Dhar M., Rafi M., Kinsella J. (2017). Transperineal template-guided prostate saturation biopsies in men with suspicion of prostate cancer: a pilot study from Pakistan. Malays J Pathol.

[63] Merrick G.S., Galbreath R.W., Bennett A., Butler W.M., Amamovich E. (2017). Incidence, grade and distribution of prostate cancer following transperineal template-guided mapping biopsy in patients with atypical small acinar proliferation. World J Urol.

[64] Merrick G.S., Irvin S., Fiano R., Anderson R., Butler W.M., Adamovich E. (2016). Pathology and quality of life outcomes following office-based transperineal prostate biopsy. Urology.

[65] Merrick G.S., Tennant A., Fiano R. (2020). Active surveillance outcomes in prostate cancer patients: the use of transperineal template-guided mapping biopsy for patient selection. World J Urol.

[66] Meyer A.R., Joice G.A., Schwen Z.R., Partin A.W., Allaf M.E., Gorin M.A. (2018). Initial experience performing in-office ultrasound-guided transperineal prostate biopsy under local anesthesia using the PrecisionPoint transperineal access system. Urology.

[67] Miah S., Eldred-Evans D., Simmons L.A.M. (2018). Patient reported outcome measures for transperineal template prostate mapping biopsies in the PICTURE study. J Urol.

[68] Miah S., Servian P., Patel A. (2020). A prospective analysis of robotic targeted MRI-US fusion prostate biopsy using the centroid targeting approach. J Robot Surg.

[69] Miller J., Perumalla C., Heap G. (2005). Complications of transrectal versus transperineal prostate biopsy. ANZ J Surg.

[70] Mischinger J., Kaufmann S., Russo G.I. (2018). Targeted vs systematic robot-assisted transperineal magnetic resonance imaging-transrectal ultrasonography fusion prostate biopsy. BJU Int.

[71] Muthuveloe D., Telford R., Viney R., Patel P. (2016). The detection and upgrade rates of prostate adenocarcinoma following transperineal template-guided prostate biopsy—a tertiary referral centre experience. Cent European J Urol.

[72] Nakai Y., Tanaka N., Anai S. (2017). Transperineal template-guided saturation biopsy aimed at sampling one core for each milliliter of prostate volume: 103 cases requiring repeat prostate biopsy. BMC Urol.

[73] Namekawa T., Fukasawa S., Komaru A. (2015). Prospective evaluation of the safety of transrectal ultrasound-guided transperineal prostate biopsy based on adverse events. Int J Clin Oncol.

[74] Novella G., Ficarra V., Galfano A. (2003). Pain assessment after original transperineal prostate biopsy using a coaxial needle. Urology.

[75] Pal R.P., Elmussareh M., Chanawani M., Khan M.A. (2012). The role of a standardized 36 core template-assisted transperineal prostate biopsy technique in patients with previously negative transrectal ultrasonography-guided prostate biopsies. BJU Int.

[76] Patel M.I., Muter S., Vladica P., Gillatt D. (2020). Robotic-assisted magnetic resonance imaging ultrasound fusion results in higher significant cancer detection compared to cognitive prostate targeting in biopsy naive men. Transl Androl Urol.

[77] Pepdjonovic L., Tan G.H., Huang S. (2017). Zero hospital admissions for infection after 577 transperineal prostate biopsies using single-dose cephazolin prophylaxis. World J Urol.

[78] Pepe P., Aragona F. (2014). Prostate biopsy: results and advantages of the transperineal approach—twenty-year experience of a single center. World J Urol.

[79] Pepe P., Cimino S., Garufi A. (2016). Detection rate for significant cancer at confirmatory biopsy in men enrolled in Active Surveillance protocol: 20 cores vs 30 cores vs MRI/TRUS fusion prostate biopsy. Arch Ital Urol Androl.

[80] Pepe P., Cimino S., Garufi A. (2017). Confirmatory biopsy of men under active surveillance: extended versus saturation versus multiparametric magnetic resonance imaging/transrectal ultrasound fusion prostate biopsy. Scand J Urol.

[81] Pepe P., Garufi A., Priolo G.D., Galia A., Fraggetta F., Pennisi M. (2018). Is it time to perform only magnetic resonance imaging targeted cores? Our experience with 1,032 men who underwent prostate biopsy. J Urol.

[82] Pepe P., Garufi A., Priolo G.D., Pennisi M. (2017). Multiparametric MRI/TRUS fusion prostate biopsy: advantages of a transperineal approach. Anticancer Res.

[83] Pepe P., Pennisi M. (2016). Erectile dysfunction in 1050 men following extended (18 cores) vs saturation (28 cores) vs saturation plus MRI-targeted prostate biopsy (32 cores). Int J Impot Res.

[84] Pepe P., Pennisi M., Fraggetta F. (2015). Anterior prostate biopsy at initial and repeat evaluation: is it useful to detect significant prostate cancer?. Int Braz J Urol.

[85] Pepe P., Pennisi M., Fraggetta F. (2020). How many cores should be obtained during saturation biopsy in the era of multiparametric magnetic resonance? Experience in 875 patients submitted to repeat prostate biopsy. Urology.

[86] Pinkstaff D.M., Igel T.C., Petrou S.P., Broderick G.A., Wehle M.J., Young P.R. (2005). Systematic transperineal ultrasound-guided template biopsy of the prostate: three-year experience. Urology.

[87] Ristau B.T., Allaway M., Cendo D. (2018). Free-hand transperineal prostate biopsy provides acceptable cancer detection and minimizes risk of infection: evolving experience with a 10-sector template. Urol Oncol.

[88] Roberts M.J., Macdonald A., Ranasinghe S. (2021). Transrectal versus transperineal prostate biopsy under intravenous anaesthesia: a clinical, microbiological and cost analysis of 2048 cases over 11 years at a tertiary institution. Prostate Cancer Prostatic Dis.

[89] Saito K., Washino S., Nakamura Y. (2017). Transperineal ultrasound-guided prostate biopsy is safe even when patients are on combination antiplatelet and/or anticoagulation therapy. BMC Urol.

[90] Salagierski M., Kania P., Wierzcholowski W., Pozniak-Balicka R. (2019). The role of a template-assisted cognitive transperineal prostate biopsy technique in patients with benign transrectal prostate biopsies: a preliminary experience. Cent European J Urol.

[91] Sigle A., Suarez-Ibarrola R., Pudimat M. (2021). Safety and side effects of transperineal prostate biopsy without antibiotic prophylaxis. Urol Oncol.

[92] Simmons L.A.M., Kanthabalan A., Arya M. (2019). Prostate Imaging Compared to Transperineal Ultrasound-guided biopsy for significant prostate cancer Risk Evaluation (PICTURE): a prospective cohort validating study assessing Prostate HistoScanning. Prostate Cancer Prostatic Dis.

[93] Singh P.B., Anele C., Dalton E. (2014). Prostate cancer tumour features on template prostate-mapping biopsies: implications for focal therapy. Eur Urol.

[94] Sivaraman A., Sanchez-Salas R., Ahmed H.U. (2015). Clinical utility of transperineal template-guided mapping biopsy of the prostate after negative magnetic resonance imaging-guided transrectal biopsy. Urol Oncol.

[95] Smith J.B., Popert R., Nuttall M.C., Vyas L., Kinsella J., Cahill D. (2014). Transperineal sector prostate biopsies: a local anesthetic outpatient technique. Urology.

[96] Song W., Kang M., Jeong B.C. (2019). The clinical utility of transperineal template-guided saturation prostate biopsy for risk stratification after transrectal ultrasound-guided biopsy. Investig Clin Urol.

[97] Stefanova V., Buckley R., Flax S. (2019). Transperineal prostate biopsies using local anesthesia: experience with 1,287 patients. prostate cancer detection rate, complications and patient tolerability. J Urol.

[98] Suzuki M., Kawakami S., Asano T. (2009). Safety of transperineal 14-core systematic prostate biopsy in diabetic men. Int J Urol.

[99] Symons J.L., Huo A., Yuen C.L. (2013). Outcomes of transperineal template-guided prostate biopsy in 409 patients. BJU Int.

[100] Szabo R.J. (2021). Free-hand transperineal prostate biopsy under local anesthesia in the office without antibiotic prophylaxis: experience with 304 cases. J Endourol.

[101] Taira A.V., Merrick G.S., Bennett A. (2013). Transperineal template-guided mapping biopsy as a staging procedure to select patients best suited for active surveillance. Am J Clin Oncol.

[102] Taira A.V., Merrick G.S., Galbreath R.W. (2010). Performance of transperineal template-guided mapping biopsy in detecting prostate cancer in the initial and repeat biopsy setting. Prostate Cancer Prostatic Dis.

[103] Takenaka A., Hara R., Ishimura T. (2008). A prospective randomized comparison of diagnostic efficacy between transperineal and transrectal 12-core prostate biopsy. Prostate Cancer Prostatic Dis.

[104] Thurtle D., Starling L., Leonard K., Stone T., Gnanapragasam V.J. (2018). Improving the safety and tolerability of local anaesthetic outpatient transperineal prostate biopsies: a pilot study of the CAMbridge PROstate Biopsy (CAMPROBE) method. J Clin Urol.

[105] Togo Y., Kubo T., Taoka R. (2014). Occurrence of infection following prostate biopsy procedures in Japan: Japanese Research Group for Urinary Tract Infection (JRGU)—a multi-center retrospective study. J Infect Chemother.

[106] Tsivian M., Abern M.R., Qi P., Polascik T.J. (2013). Short-term functional outcomes and complications associated with transperineal template prostate mapping biopsy. Urology.

[107] Voss J., Pal R., Ahmed S., Hannah M., Jaulim A., Walton T. (2018). Utility of early transperineal template-guided prostate biopsy for risk stratification in men undergoing active surveillance for prostate cancer. BJU Int.

[108] Vyas L., Acher P., Kinsella J. (2014). Indications, results and safety profile of transperineal sector biopsies (TPSB) of the prostate: a single centre experience of 634 cases. BJU Int.

[109] Wadhwa K., Carmona-Echeveria L., Kuru T. (2017). Transperineal prostate biopsies for diagnosis of prostate cancer are well tolerated: a prospective study using patient-reported outcome measures. Asian J Androl.

[110] Wajswol E., Winoker J.S., Anastos H. (2020). A cohort of transperineal electromagnetically tracked magnetic resonance imaging/ultrasonography fusion-guided biopsy: assessing the impact of inter-reader variability on cancer detection. BJU Int.

[111] Wang L., Wang X., Zhao W. (2019). Surface-projection-based transperineal cognitive fusion targeted biopsy of the prostate: an original technique with a good cancer detection rate. BMC Urol.

[112] Wegelin O., Exterkate L., van der Leest M. (2019). Complications and adverse events of three magnetic resonance imaging-based target biopsy techniques in the diagnosis of prostate cancer among men with prior negative biopsies: results from the FUTURE trial, a multicentre randomised controlled trial. Eur Urol Oncol.

[113] Wetterauer C., Shahin O., Federer-Gsponer J.R. (2020). Feasibility of freehand MRI/US cognitive fusion transperineal biopsy of the prostate in local anaesthesia as in-office procedure-experience with 400 patients. Prostate Cancer Prostatic Dis.

[114] Yamamoto S., Kin U., Nakamura K. (2005). Transperineal ultrasound-guided 12-core systematic biopsy of the prostate for patients with a prostate-specific antigen level of 2.5-20 ng/ml in Japan. Int J Clin Oncol.

[115] Yang X., Lee A.Y., Law Y.M. (2020). Stereotactic robot-assisted transperineal prostate biopsy under local anaesthesia and sedation: moving robotic biopsy from operating theatre to clinic. J Robot Surg.

[116] Yazici S., Kiziloz H., Bozaci A.C., Baydar D.E., Del Biondo D., Ozen H. (2016). Predictors of prostate cancer in ultrasound-guided transperineal saturation biopsy in Turkish men with multiple prior negative biopsies. Urologia.

[117] Young R., Norris B., Reeves F., Peters J.S. (2019). A retrospective comparison of transrectal and transperineal prostate biopsies: experience of a single surgeon. J Endourol.

[118] Zhang F., Shao Q., Du Y., Tian Y. (2019). Evaluation of 24-core coaxial needle saturation biopsy of the prostate by the transperineal approach in detecting prostate cancer in patients without previous biopsy history: a single-center report. J Cancer Res Ther.

[119] Cohen J.E., Landis P., Trock B.J. (2015). Fluoroquinolone resistance in the rectal carriage of men in an active surveillance cohort: longitudinal analysis. J Urol.

[120] Cook I., Angel J.B., Vera P.L., Demos J., Preston D. (2015). Rectal swab testing before prostate biopsy: experience in a VA Medical Center urology practice. Prostate Cancer Prostatic Dis.

[121] Adibi M., Pearle M.S., Lotan Y. (2012). Cost-effectiveness of standard vs intensive antibiotic regimens for transrectal ultrasonography (TRUS)-guided prostate biopsy prophylaxis. BJU Int.

[122] Kuula L.S.M., Viljemaa K.M., Backman J.T., Blom M. (2019). Fluoroquinolone-related adverse events resulting in health service use and costs: a systematic review. PLoS One.

[123] Block M, Blanchard DL. Aminoglycosides. In: StatPearls [Internet]. Treasure Island, FL: StatPearls Publishing; 2021. https://www.ncbi.nlm.nih.gov/books/NBK541105/

[124] Berger A.P., Gozzi C., Steiner H. (2004). Complication rate of transrectal ultrasound guided prostate biopsy: a comparison among 3 protocols with 6, 10 and 15 cores. J Urol.

[125] Wagenlehner F.M., van Oostrum E., Tenke P. (2013). Infective complications after prostate biopsy: outcome of the Global Prevalence Study of Infections in Urology (GPIU) 2010 and 2011, a prospective multinational multicentre prostate biopsy study. Eur Urol.

[126] Roberts M.J., Bennett H.Y., Harris P.N. (2017). Prostate biopsy-related infection: a systematic review of risk factors, prevention strategies, and management approaches. Urology.

[127] Carignan A., Roussy J.F., Lapointe V., Valiquette L., Sabbagh R., Pepin J. (2012). Increasing risk of infectious complications after transrectal ultrasound-guided prostate biopsies: time to reassess antimicrobial prophylaxis?. Eur Urol.

