# Supplementary material for: Role of Prophylactic Antibiotics in Transperineal Prostate Biopsy: A Systematic Review and Meta-analysis
Source: Eur Urol Open Sci. 2022 Jan 29;37:53–63. doi: 10.1016/j.euros.2022.01.001 (PMC8883190; doi:10.1016/j.euros.2022.01.001)
Supplement: Supplementary data 1 [file mmc1.docx]

**Supplementary Figure 1.** Meta-analysis for the proportion of men who underwent transperineal prostate biopsy without receiving periprocedural prophylactic antibiotics and developed postprocedural non-septic infections. I^2^=71.6%, p<0.01.

**Supplementary Figure 2.** Meta-analysis for the proportion of men who underwent transperineal prostate biopsy without receiving periprocedural prophylactic antibiotics and developed postprocedural either septic or non-septic infections. I^2^=71.3%, p<0.01.

**Supplementary Figure 3.** Meta-analysis for the proportion of men who underwent transperineal prostate biopsy after receiving periprocedural prophylactic antibiotics and developed postprocedural sepsis. I^2^=0.0%, p=0.998.

**Supplementary Figure 4.** Meta-analysis for the proportion of men who underwent transperineal prostate biopsy after receiving periprocedural prophylactic antibiotics and developed postprocedural non-septic infections. I^2^=86.8%, p<0.01.

**Supplementary Figure 5.** Meta-analysis for the proportion of men who underwent transperineal prostate biopsy after receiving periprocedural prophylactic antibiotics and developed postprocedural either septic or non-septic infections. I^2^=86.1%, p<0.01.
