# Supplementary figures and images for: Role of Prophylactic Antibiotics in Transperineal Prostate Biopsy: A Systematic Review and Meta-analysis
Source: Eur Urol Open Sci. 2022 Jan 29;37:53–63. doi: 10.1016/j.euros.2022.01.001 (PMC8883190; doi:10.1016/j.euros.2022.01.001)

FIGURE S1

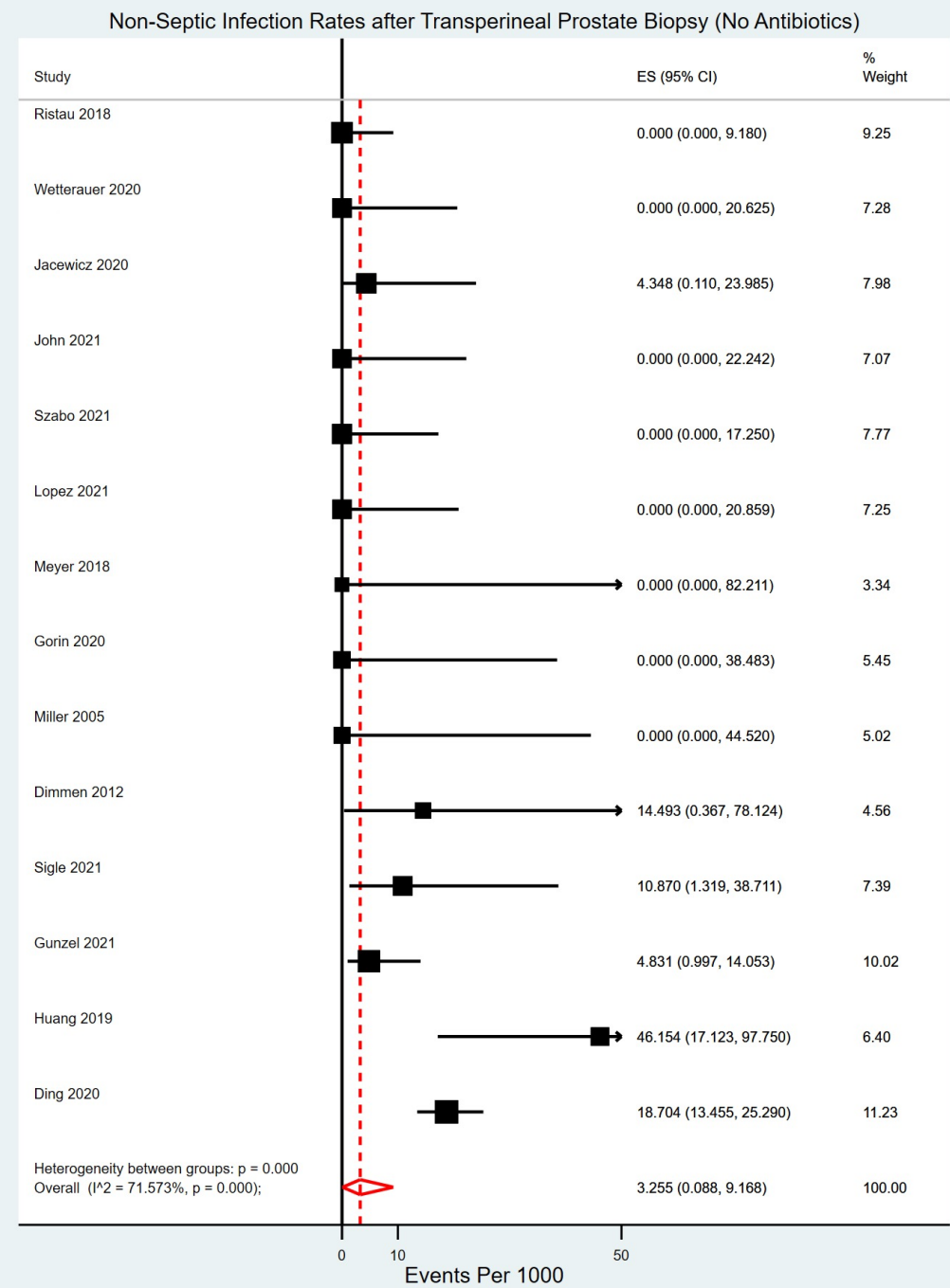

FIGURE S2

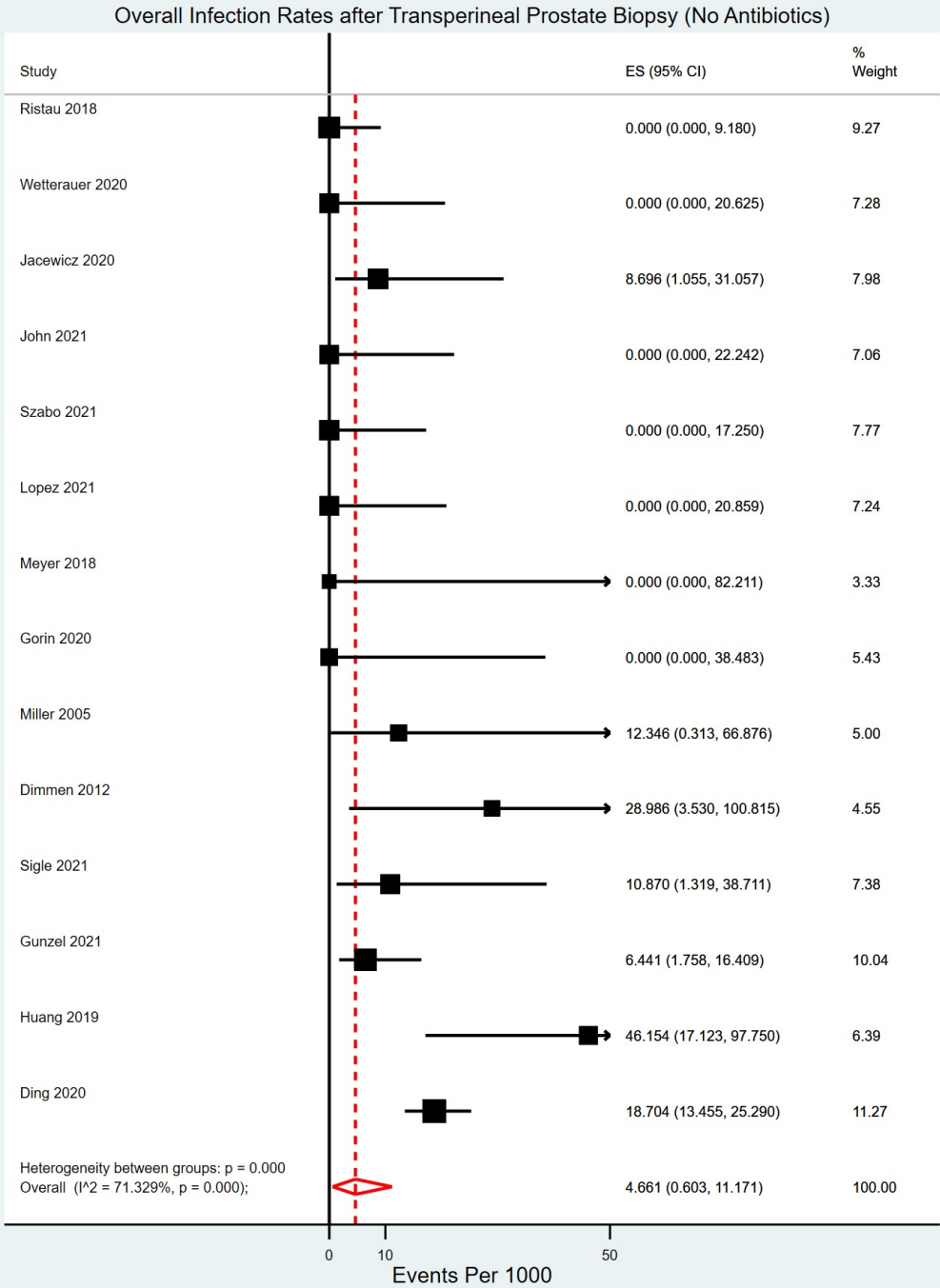

**FIGURE S3**

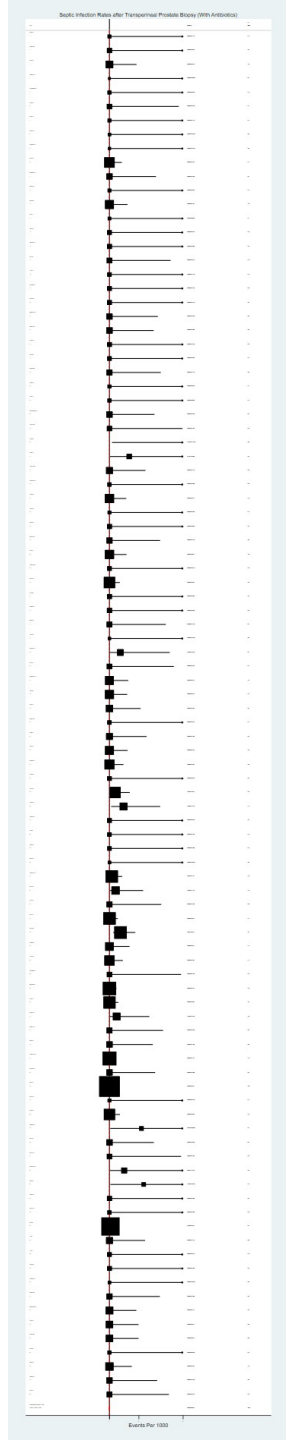

## FIGURE S4

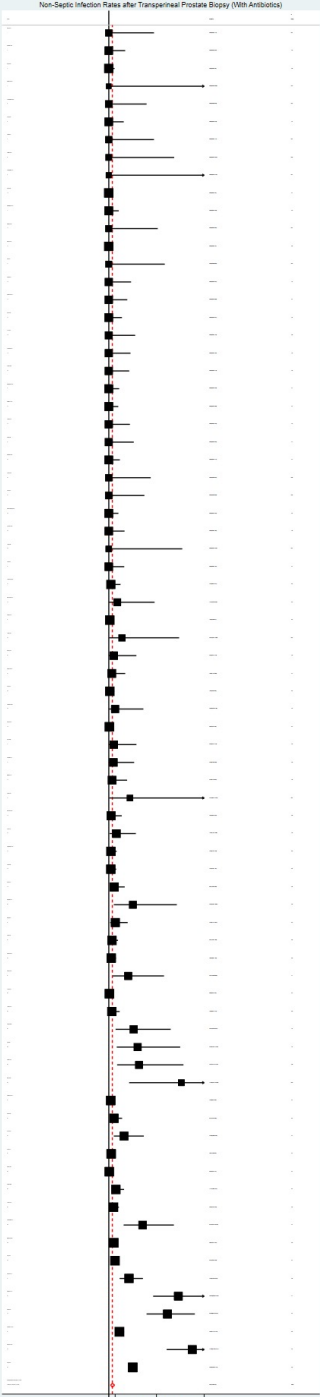

FIGURE S5

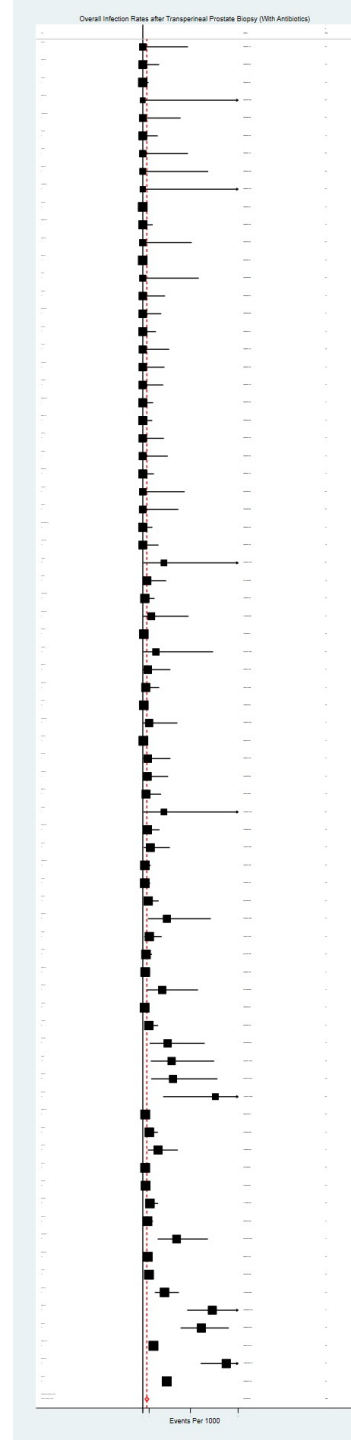

Supplement: Supplementary data 2 [file mmc2.pdf]
