# Supplementary material for: Role of Prophylactic Antibiotics in Transperineal Prostate Biopsy: A Systematic Review and Meta-analysis
Source: Eur Urol Open Sci. 2022 Jan 29;37:53–63. doi: 10.1016/j.euros.2022.01.001 (PMC8883190; doi:10.1016/j.euros.2022.01.001)
Supplement: Supplementary data 3 [file mmc3.docx]

**Supplementary Table 1. Summary data for studies with men undergoing transperineal prostate biopsy with prophylactic antibiotics.**

| **Study name** | **Country** | **Sample Size, n** | **Type of Study** | **Age (years)** | **Mean or Median Number of Biopsy cores, n** | **Anesthesia** | **Antibiotic regimen** | **Duration of Antibiotic (days)** | **Sepsis, n** | **Non-septic Infections, n** | **All Infections, n** |
| --- | --- | --- | --- | --- | --- | --- | --- | --- | --- | --- | --- |
| Pepe et al. (2017) [1] | Italy | 49 | Cohort | Median 66  (IQR 63-68) | NR | Sedation only | NR | NR | 0 | NR | NR |
| Klatte et al. (2013) [2] | Austria | 50 | Cohort | Median 57.5  (IQR 10) | 24 | NR | Ciprofloxacin | 3 | 0 | 0 | 0 |
| Pepe et al. (2018) [3] | Italy | 1032 | Cohort | Median 63  (Range 47-78) | 30 | Sedation only | Levofloxacin | 3 | 0 | NR | NR |
| Emiliozzi et al. (2001) [4] | Italy | 141 | Cohort | Mean 67  (Range 50-82) | 12 | Local only | Fluoroquinolone | 3 | 0 | 0 | 0 |
| Pepe et al. (2015) [5] | Italy | 400 | Cohort | Median 63.5  (Range 47-75) | 27 | Sedation only | Levofloxacin | 3 | 0 | 0 | 0 |
| Yamamoto et al. (2005) [6] | Japan | 300 | Cohort | Median 69  (Range 38-88) | 12 | General | Sulbactam sodium/ Cefoperazone sodium (Days 1-2) Levofloxacin (Days 3-7) | 7 | 0 | 1 | 1 |
| Sivaraman et al. (2015) [7] | UK | 75 | Cohort | Mean 61.6  (SD 6.5) | 31.2 | General | NR | NR | 0 | 1 | 1 |
| Mehmood et al. (2017) [8] | Pakistan | 16 | Cohort | Mean 67.8  (Range 55-84) | NR | General | NR | 1 | 0 | 0 | 0 |
| Mabjeesh et al. (2012) [9] | Israel | 92 | Cohort | Mean 63.8  (SD 5.8) | 30 | General or Spinal | Ciprofloxacin or Ofloxacin | 3 | 1 | NR | NR |
| Kuru et al. (2013) [10] | Germany | 347 | Cohort | Mean 65.3 (Range 42-82) | 24 | General | Fluoroquinolone | 3 | 0 | 3 | 3 |
| Kum et al. (2019) [11] | UK | 243 | Cohort | Mean 66.2  (Range 41-80) | 33 | General or Spinal | Gentamicin and co-amoxiclav | NR | 0 | NR | NR |
| Bennett et al. (2017) [12] | Spain | 60 | Cohort | Mean 64.1  (SD 6.7) | 13.8 | Local only | NR | NR | 0 | 0 | 0 |
| Ekwueme et al. (2013) [13] | UK | 270 | Cohort | Median 56.4  (Range 43-85) | 28 | General | Gentamicin and Metronidazole single-dose Ciprofloxacin x 5 days | 5 | 1 | 1 | 2 |
| Bigliocchi et al. (2007) [14] | Italy | 6 cores: 836 12 cores: 315 | Cohort | Mean 68 (SD 7.8) Mean 67.5 (SD 8.2) | NR | Local only | Fluoroquinolone | 4 | 0 | 12 | 12 |
| Zhang et al. (2019) [15] | China | 644 | Cohort | Mean 70.5  (Range 61-82) | NR | General | Levofloxacin | Single-dose | 0 | 1 | 1 |
| Young et al. (2019) [16] | Australia | 417 | RCT | Mean 63.8  (Range 39-90) | 20.5 | General | Gentamicin, Amoxicillin or other | Single-dose | 2 | 2 | 4 |
| Yazici et al. (2016) [17] | Turkey | 48 | Cohort | Median 61.5  (Range 46-79) | 54 | General | NR | 3 | 0 | 1 | 1 |
| Yang et al. (2020) [18] | Singapore | 30 | Cohort | Median 66  (IQR 53-80) | 30 | Local and sedation | Co-amoxiclav | 3 | 1 | 0 | 1 |
| Wang et al. (2019) [19] | China | 126 | Cohort | Mean 68.7  (SD 9.2) | 14.5 | General or Spinal | NR | Single-dose | 0 | 1 | 1 |
| Wajswol et al. (2020) [20] | US | 213 | Cohort | Median 67.5  (Range 44-89) | NR | Local only | Gentamicin | Single-dose | 0 | 1 | 1 |
| Wadhwa et al. (2017) [21] | UK, Germany | 201 | Cohort | Mean 64.4  (SD 7.54) | 27.3 | General | NR | NR | 0 | 22 | 22 |
| Vyas et al. (2014) [22] | UK | Prior negative: 174 Biopsy naïve: 153 Prior GS 6: 307 | Cohort | Median 63  (Range 42-80) Median 62  (Range 38-81) Median 64  (Range 43-81) | 31 | General or Local only | Amikacin single-dose Ciprofloxacin x 3 days | 3 | 0 | 1 | 1 |
| Voss et al. (2018) [23] | UK | 208 | Cohort | Mean 63.5  (Range 45-77) | 50 | General or Local only | NR | NR | 0 | 5 | 5 |
| Takenaka et al. (2008) [24] | Japan | 100 | RCT | Mean 71.1  (SD 7.53) | 12 | Local only | Levofloxacin | 1 | 0 | 1 | 1 |
| Wegelin et al. (2019) [25] | Netherlands | 79 | RCT | Mean 64.6  (SD 6.9) | 14 | General or Spinal | Ciprofloxacin | 3 | 0 | 3 | 3 |
| Symons et al. (2013) [26] | Australia | 409 | Cohort | Mean 63.3  (95% CI 0.8) | 19.2 | General | Gentamicin single-dose Norfloxacin x 3 days | 3 | 1 | 13 | 14 |
| Suzuki et al. (2009) [27] | Japan | Diabetic: 135 Non-diabetic: 404 | Cohort | Median 70  (IQR 52-86) Median 71  (IQR 43-87) | NR | General or Sedation only or Spinal | Cephalosporin or Fluoroquinolone | 1-3 | 0 0 | 2 4 | 2 4 |
| Stefanova et al. (2019) [28] | UK | 1287 | RCT | Median 66  (Range 38-92) | NR | Local only | Cefuroxime, Cephalexin, or Trimethoprim/ Sulfamethoxazole | Single-dose | 1 | 4 | 5 |
| Song et al. (2019) [29] | Korea | 155 | Cohort | Median 65  (Range 47-79) | 36 | General | Cephalosporin or Fluoroquinolone and Amikacin | 7 | 0 | 0 | 0 |
| Smith et al. (2014) [30] | UK | 50 | Cohort | Median 63  (Range 47-79) | 45 | Local only | Aminoglycoside and Fluoroquinolone | 3 | 0 | 0 | 0 |
| Togo et al. (2014) [31] | Japan | 1234 | RCT | Median 69  (Range 40-98) | NR | NR | Multiple | 1-7 | 0 | 12 | 12 |
| Thurtle et al. (2018) [32] | UK | 34 | Cohort | Median 71.5  (Range 49-77) | 11 | Local only | Ciprofloxacin | Single-dose | 0 | 0 | 0 |
| Singh et al. (2014) [33] | UK | 291 | Cohort | Median 61 (Range 40-81) | 29 | NR | Cefuroxime, Gentamicin, and Metronidazole | Single-dose | 0 | 3 | 3 |
| Simmons et al. (2019) [34] | UK | 236 | Cohort | Mean 62.1  (Range 41.7-83.2) | 48.7 | General or Spinal | NR | NR | 0 | 31 | 31 |
| Salagierski et al. (2019) [35] | Poland | 22 | Cohort | Median 67  (Range 56-75) | 24 | General | Ciprofloxacin | 5 | 0 | 0 | 0 |
| Saito et al. (2017) [36] | Japan | Control: 449 Antiplatelet/ Anticoag: 149 | Cohort | Mean 68.7 (SD 7.5) Mean 71.6 (SD 6.7) | 16 | General or Spinal | Levofloxacin | 3 | 0 | 3 | 3 |
| Pepe et al. (2020) [37] | Italy | 875 | Cohort | Median 63  (Range 47-78) | 18 | Sedation only | Cefazolin | Single-dose | 0 | 0 | 0 |
| Szabo et al. (2021) [38]* | US | 272 | Cohort | Median 63  (Range 29–93) | 20 | Local only | Ceftriaxone or Ciprofloxacin (n=30, 12%) | Single-dose | 0 | 0 | 0 |
| Pepe et al. (2016) [39] | Italy | 1150 | Cohort | Median 63  (Range 56-71) | NR | Sedation only | Levofloxacin | 3 | 0 | 1 | 1 |
| Pepe et al. (2017) [40] | Italy | 150 | Cohort | Median 62  (Range 47-78) | NR | Sedation only | NR | NR | 0 | NR | NR |
| Muthuveloe et al. (2016) [41] | UK | 200 | Cohort | Median 67  (Range 41-81) | 24 | General | Gentamicin and Metronidazole single-dose Ciprofloxacin x 3 days | 3 | 1 | NR | NR |
| Mischinger et al. (2018) [42] | Germany | 232 | Cohort | Median 66  (IQR 60-73) | NR | General | NR | NR | 0 | 0 | 0 |
| Miah et al. (2020) [43] | UK | 86 | Cohort | Median 63.5  (IQR 10.6) | 28 | General | Gentamicin | Single-dose | 1 | NR | NR |
| Merrick et al. (2016) [44] | US | 46 | Cohort | Median 63.5 | 18 | Local only | Ciprofloxacin | Single-dose | 0 | 0 | 0 |
| Merrick et al. (2017) [45] | US | 132 | Cohort | Mean 63.8,  Median 65 | 58 | General | NR | NR | 0 | NR | NR |
| Roberts et al. (2020) [46] | Australia | 774 | Cohort | Mean 65.1 | 22 | General | Ciprofloxacin | Single-dose | 0 | 3 | 3 |
| Ristau et al. (2018) [47]* | US | 600 | Cohort | Median 68  (IQR 61-74) | 16 | Local and sedation | Cephalexin (60%) | Single-dose | 0 | 0 | 0 |
| Pepe et al. (2016) [48] | Italy | 75 | Cohort | Median 66  (Range 58-73) | 30 | Sedation only | NR | NR | 0 | NR | NR |
| Pepe et al. (2014) [49] | Italy | 12 cores: 1428 18 cores: 1330 >24 cores: 630 Total: 4000 | Cohort | Median 63.2  (Range 40-73) Median 61.8  (Range 49-74) Median 58.2  (Range 48-72) | NR | Local only or Sedation only | Levofloxacin | 3 | 0 0 0 0 | 65 55 31 174 | 65 55 31 174 |
| Pal et al. (2012) [50] | UK | 40 | Cohort | Mean 62.9  (Range 49-73) | 26 | General | Co-amoxiclav and Gentamicin | Single-dose | 0 | 0 | 0 |
| Novella et al. (2003) [51] | Italy | TruGuide: 51 Conventional: 51 | RCT | Mean 63.7  (SD 7.14) Mean 65.9  (SD 7.54) | NR | Local only | Trimethoprim/ Sulfamethoxazole or Fluoroquinolone | Single-dose | 0 | 0 4 | 0 4 |
| Namekawa et al. (2015) [52] | Japan | 1663 | Cohort | Median 68  (Range 30-86) | NR | Spinal | Sulbactam/ Cefoperazone | 3 | 0 | 28 | 28 |
| Nakai et al. (2017) [53] | Japan | 103 | Cohort | Median 69  (Range 37-83) | 37 | General | Cefazolin | Single-dose | 0 | 0 | 0 |
| Martorana et al. (2015) [54] | Italy | 125 | Cohort | Mean 64.73  (Range 52-81) | 32 | Sedation only | Ciprofloxacin | Single-dose | 0 | 0 | 0 |
| Marra et al. (2020) [55] | Italy, China | 1327 | Cohort | Mean 66.8  (SD 7.4) | 15.3 | Local only | Ciprofloxacin or Cefazolin | Single-dose | 5 | 1 | 6 |
| Mai et al. (2016) [56] | China | 3007 | Cohort | Mean 69.1  (Range 30-91) | 19.3 | Local only | Levofloxacin | 3 | 1 | NR | NR |
| Li et al. (2007) [57] | China | 303 | Cohort | Mean 69.7  (Range 35-90) | 23.7 | Local only | NR | 3 | 0 | NR | NR |
| Kum et al. (2018) [58] | London | 176 | Cohort | Mean 65  (Range 36-83) | 31 | Local and sedation | Gentamicin | Single-dose | 0 | 0 | 0 |
| Losa et al. (2013) [59] | Italy | 87 | Cohort | Median 63.9  (Range 46-78) | 40 | Spinal | Cefoxitin | Single-dose | 0 | 0 | 0 |
| Lo et al. (2019) [60] | China | 100 | Cohort | Mean 67.7  (SD 6.2) | NR | Local only | Co-amoxiclav and Ciprofloxacin | 1 | 0 | NR | NR |
| Hadaschik et al. (2011) [61] | Germany | 106 | Cohort | Median 66  (Range 42-83) | 24 | General | Fluoroquinolone | Single-dose | 0 | 0 | 0 |
| Guo et al. (2015) [62] | China | 167 | RCT | Mean 67.2 (SD 6.8) | 11.1 | Local only | Ciprofloxacin | 2 | 0 | 2 | 2 |
| Guo et al. (2017) [63] | China | 1300 | Cohort | Mean 70.5  (Range 34-89) | 14.5 | Local only | Ciprofloxacin and Metronidazole | 1 | 0 | 5 | 5 |
| Iremashvili et al. (2010) [64] | Russia | Local: 75 Local + nerve block: 75 | RCT | Mean 69.09  (SD 7.87) Mean 68.32  (SD 7.35) | 12 | Local only and Nerve block | Ciprofloxacin | 3 | 0 | 8 | 8 |
| Igel et al. (2001) [65] | USA | 88 | Cohort | Mean 65  (Range 54-79) | 17 | General or Local only | Fluoroquinolone | 3 | 0 | 4 | 4 |
| Huang et al. (2015) [66] | Australia | Active surveillance: 40 Prior negative: 54 Other: 17 | Cohort | Mean 63.6  (Range 47-73) Mean 60.5  (Range 45-74) Mean 65  (Range 53-78) | NR | General | Cefazolin | Single-dose | 0 | NR | NR |
| Hara et al. (2008) [67] | Japan | 126 | RCT | Mean 71 (SD 7.29) | 12 | Spinal | Levofloxacin | Single-dose | 0 | 1 | 1 |
| Gershman et al. (2013) [68] | USA | 34 | Cohort | Mean: 66.2  (SD 6.4) | 25.8 | General | NR | NR | 0 | NR | NR |
| Furuno et al. (2004) [69] | Japan | 113 | Cohort | Mean 64.5  (Range 50-81) | 18 | General or Spinal | Fluoroquinolone | 5 | 0 | 0 | 0 |
| Emiliozzi et al. (2004) [70] | Italy | 6 cores: 107 12 cores: 107 | RCT | Median 67 Median 68 | NR | Local only | Fluoroquinolone | 3 | 0 | NR | NR |
| Eldred et al. (2016) [71] | UK | 402 | Cohort | Mean 61.1 (SD 8.7) | 28.6 | General or Local only | Aminoglycoside single-dose Fluoroquinolone x 3-5 days | 3-5 | 0 | NR | NR |
| Taira et al. (2010) [72] | USA | 373 | Cohort | Median: 64.2 | 57 | General | NR | NR | 0 | NR | NR |
| Wetterauer et al. (2020) [73]* | Switzerland | 400 | Cohort | Median 66  (Range 49-86) | 13 | Local only | Fluoroquinolone the night before + 1 hour pre-op (29.5%) Fluoroquinoline 1 hour pre-op (26.2%) None (44.3%) | 0-1 | 0 | 0 | 0 |
| DiBianco et al. (2016) [74] | USA | 244 | Cohort | Mean 66.7  (SD 8.47) | 14.4 | Local and sedation | Cefazolin | Single-dose | 0 | 0 | 0 |
| Demura et al. (2005) [75] | Japan | 371 | Cohort | Mean 67.4  (Range 43-87) | 20.1 | Spinal | Fluoroquinolone | 5 | 0 | NR | NR |
| Danforth et al. (2012) [76] | USA | 137 | Cohort | Mean 70  (Range 59-82) | NR | General | Ciprofloxacin | 8 | 0 | 1 | 1 |
| Bittner et al. (2015) [77] | USA | 191 | Cohort | Median 64.6 | 54 | General | Levofloxacin | 5 | 0 | 1 | 1 |
| Cronin et al. (2017) [78] | UK | 109 | Cohort | Mean 65  (Range 46-84) | 20 | General | Co-amoxiclav | Single-dose | 0 | 0 | 0 |
| Bott et al. (2006) [79] | UK | 60 | Cohort | Mean 64 (SD 6.4) | 24 | General | NR | NR | 0 | NR | NR |
| Bittner et al. (2013) [80] | USA | 485 | Cohort | Median 65, Mean 64.8 | 55.6 | General | NR | NR | 0 | NR | NR |
| Merrick et al. (2020) [81] | USA | 226 | Cohort | Mean 67.5 | 59.5 | General | NR | NR | 0 | NR | NR |
| Patel et al. (2020) [82] | Australia | 92 | Cohort | Median 63  (IQR 58-68) | NR | General | NR | NR | 0 | 0 | 0 |
| Tsivian et al. (2013) [83] | USA | 84 | Cohort | Median 66.6  (IQR 59.4-69.5) | 56 | NR | NR | Single-dose | 0 | 4 | 4 |
| Pepdjonovic et al. (2017) [84] | Australia | 577 | Cohort | Median 65  (Range 41-87) | 24.4 | General | Cephazolin | Single-dose | 0 | 2 | 2 |
| Huang et al. (2016) [85] | China | 98 | Cohort | Mean 63.4 (SD 9.81) | 15.2 | General | Cefuroxime | Single-dose | 0 | 3 | 3 |
| Pinkstaff et al. (2005) [86] | USA | 210 | Cohort | Mean 66.3  (Range 46-81) | 21.2 | General | Fluoroquinolone | 3 | 0 | 0 | 0 |
| Chiu et al. (2020) [87] | China | 611 | Cohort | Median 69  (IQR 65-72) | 20 | Local only | Co-amoxiclav and Ciprofloxacin | Single-dose | 0 | 2 | 2 |
| Cerruto et al. (2014) [88] | Italy | 54 | RCT | Mean 66.5  (SD 8.87) | 14 | Local only | Prulifloxacin | 3 | 0 | 0 | 0 |
| Taira et al. (2013) [89] | USA | 64 | Cohort | Mean 68.1 (SD 13.5) | 58.5 | General | NR | NR | 0 | 0 | 0 |
| Bass et al. (2017) [90] | UK | 181 | Cohort | Mean 68  (Range 46-92) | NR | Local only | Gentamicin | Single-dose | 0 | NR | NR |
| Miah et al. (2018) [91] | UK | 249 | Cohort | Mean 62 (SD 7) | 49 | NR | Gentamicin and Cefuroxime | Single-dose | 0 | 23 | 23 |
| Babaei et al. (2016) [92] | Iran | 240 | RCT | Mean 64 (SD 8) | NR | Local only or Nerve block | Fluoroquinolone | 4 | 0 | 0 | 0 |
| Baba et al. (2018) [93] | Japan | 485 | Cohort | Median 70  (Range 41-90) | NR | NR | Cefazolin | 1 | 1 | 4 | 5 |
| Asano et al. (2015) [94] | Japan | Local: 756 Spinal: 56 General: 3 | Cohort | Median 69  (IQR 62-74) Median 72  (IQR 67-77) Median 70  (IQR 64-74) | 14 | Local only, Spinal, or General | Levofloxacin or Tosuflocazin | 1 | 0 | 6 | 6 |
| Jacewicz et al. (2020) [95]* | Multinational | 377 | Cohort | Mean 67  (95% CI 66-68) | NR | Local only | Trimethoprim/ Sulfamethoxazole (39%) | NR | 1 | 1 | 2 |
| Bhatt et al. (2018) [96] | Ireland | 35 | Cohort | Median 61  (Range 48-73) | 20 | General | Ciprofloxacin 12 hours preop Gentamicin periop | 1 | 0 | 4 | 4 |
| Lopez et al. (2021) [97]* | UK, New Zealand, Hong Kong | 163 | Cohort | Median 68  (IQR 62-72) | 24 | Local only | Ciprofloxacin | Single-dose | 0 | 0 | 0 |
| John et al. (2021) [98]* | UK | 149 | Cohort | Median 70  (IQR 66-74) | 20 | Local only | Ciprofloxacin | Single-dose | 1 | 0 | 1 |

NR: not reported; SD: standard deviation; IQR: interquartile range; CI: confidence interval; GS: Gleason Score

**Supplementary Table 2. Summary data for studies with detailed infectious complications.**

| **Study name** | **Country** | **Sample Size, n** | **Age (years)** | **Antibiotic regimen** | **Duration of Antibiotic (days)** | **Urinary tract infection, n** | **Pyelonephritis, n** | **Epididymoorchitis, n** | **Prostatitis, n** | **Fever, n** | **Non-septic Infections, n** |
| --- | --- | --- | --- | --- | --- | --- | --- | --- | --- | --- | --- |
| Pepe et al. (2017) [1] | Italy | 49 | Median 66 (IQR 63-68) | NR | NR | NR | NR | NR | NR | NR | NR |
| Klatte et al. (2013) [2] | Austria | 50 | Median 57.5 (IQR 10) | Ciprofloxacin | 3 | 0 | 0 | 0 | 0 | 0 | 0 |
| Pepe et al. (2018) [3] | Italy | 1032 | Median 63 (Range 47-78) | Levofloxacin | 3 | NR | NR | NR | NR | NR | NR |
| Emiliozzi et al. (2001) [4] | Italy | 141 | Mean 67 (Range 50-82) | Fluoroquinolone | 3 | 0 | 0 | 0 | 0 | 0 | 0 |
| Pepe et al. (2015) [5] | Italy | 400 | Median 63.5 (Range 47-75) | Levofloxacin | 3 | 0 | 0 | 0 | 0 | 0 | 0 |
| Yamamoto et al. (2005) [6] | Japan | 300 | Median 69 (Range 38-88) | Sulbactam sodium/ Cefoperazone sodium (Days 1-2) Levofloxacin (Days 3-7) | 7 | 0 | 0 | 0 | 1 | 0 | 1 |
| Sivaraman et al. (2015) [7] | UK | 75 | Mean 61.6 (SD 6.5) | NR | NR | 0 | 0 | 0 | 1 | 0 | 1 |
| Mehmood et al. (2017) [8] | Pakistan | 16 | Mean 67.8 (Range 55-84) | NR | 1 | 0 | 0 | 0 | 0 | 0 | 0 |
| Mabjeesh et al. (2012) [9] | Israel | 92 | Mean 63.8 (SD 5.8) | Ciprofloxacin or Ofloxacin | 3 | NR | NR | NR | NR | NR | NR |
| Kuru et al. (2013) [10] | Germany | 347 | Mean 65.3 (Range 42-82) | Fluoroquinolone | 3 | 3 | 0 | 0 | 0 | 0 | 3 |
| Kum et al. (2019) [11] | UK | 243 | Mean 66.2  (Range 41-80) | Gentamicin and co-amoxiclav | NR | NR | NR | NR | NR | NR | NR |
| Bennett et al. (2017) [12] | Spain | 60 | Mean 64.1 (SD 6.7) | NR | NR | 0 | 0 | 0 | 0 | 0 | 0 |
| Ekwueme et al. (2013) [13] | UK | 270 | Median 56.4 (Range 43-85) | Gentamicin and Metronidazole single-dose Ciprofloxacin x 5 days | 5 | 0 | 0 | 0 | 1 | 0 | 1 |
| Dimmen et al. (2012) [99] | Norway | 69 | Median 64.5 (Range 50-78) | None | 0 | 1 | 0 | 0 | 0 | 0 | 1 |
| Bigliocchi et al. (2007) [14] | Italy | 6 cores: 836 12 cores: 315 | Mean 68 (SD 7.8) Mean 67.5 (SD 8.2) | Fluoroquinolone | 4 | NR | NR | NR | NR | 12 | 12 |
| Zhang et al. (2019) [15] | China | 644 | Mean 70.5 (Range 61-82) | Levofloxacin | Single-dose | NR | NR | NR | NR | 1 | 1 |
| Young et al. (2019) [16] | Australia | 417 | Mean 63.8 (Range 39-90) | Gentamicin, Amoxicillin or other | Single-dose | 2 | NR | NR | NR | NR | 2 |
| Yazici et al. (2016) [17] | Turkey | 48 | Median 61.5 (Range 46-79) | NR | 3 | 0 | 0 | 1 | 0 | 0 | 1 |
| Yang et al. (2020) [18] | Singapore | 30 | Median 66 (IQR 53-80) | Co-amoxiclav | 3 | 0 | 0 | 0 | 0 | 0 | 0 |
| Wang et al. (2019) [19] | China | 126 | Mean 68.7 (SD 9.2) | NR | Single-dose | 0 | 0 | 1 | 0 | 0 | 1 |
| Wajswol et al. (2020) [20] | US | 213 | Median 67.5 (Range 44-89) | Gentamicin | Single-dose | NR | NR | NR | NR | NR | 1 |
| Wadhwa et al. (2017) [21] | UK, Germany | 201 | Mean 64.37 (SD 7.54) | NR | NR | NR | NR | NR | NR | 22 | 22 |
| Vyas et al. (2014) [22] | UK | Prior negative: 174 Biopsy naïve: 153 Prior GS 3+3: 307 | Median 63 (Range 42-80) Median 62 (Range 38-81) Median 64 (Range 43-81) | Amikacin single-dose Ciprofloxacin x 3 days | 3 | NR | 0 | 1 | 0 | 0 | 1 |
| Voss et al. (2018) [23] | UK | 208 | Mean 63.5 (Range 45-77) | NR | NR | 1 | NR | NR | NR | NR | 5 |
| Takenaka et al. (2008) [24] | Japan | 100 | Mean 71.1 (SD 7.53) | Levofloxacin | 1 | 0 | 0 | 0 | 0 | 1 | 1 |
| Wegelin et al. (2019) [25] | Netherlands | 79 | Mean 64.6 (SD 6.9) | Ciprofloxacin | 3 | 1 | 0 | 0 | 0 | 2 | 3 |
| Symons et al. (2013) [26] | Australia | 409 | mean 63.3  (95% CI 0.8) | Gentamicin single-dose Norfloxacin x 3 days | 3 | NR | NR | NR | NR | 13 | 13 |
| Suzuki et al. (2009) [27] | Japan | Diabetic: 135 Non-diabetic: 404 | Median 70 (IQR 52-86) Median 71 (IQR 43-87) | Cephalosporin or Fluoroquinolone | 1-3 | 0 | 0 | 0 | 0 | 6 | 6 |
| Stefanova et al. (2019) [28] | UK | 1287 | Median 66 (Range 38-92) | Cefuroxime, Cephalexin, or Trimethoprim/ Sulfamethoxazole | Single-dose | 4 | 0 | 0 | 0 | 0 | 4 |
| Song et al. (2019) [29] | Korea | 155 | Median 65 (Range 47-79) | Cephalosporin or Fluoroquinolone and Amikacin | 7 | 0 | 0 | 0 | 0 | 0 | 0 |
| Smith et al. (2014) [30] | UK | 50 | Median 63 (Range 47-79) | Aminoglycoside and Fluoroquinolone | 3 | 0 | 0 | 0 | 0 | 0 | 0 |
| Togo et al. (2014) [31] | Japan | 1234 | Median 69 (Range 40-98) | Multiple | 1-7 | 6 | NR | 0 | 4 | 2 | 12 |
| Thurtle et al. (2018) [32] | UK | 34 | Median 71.5 (Range 49-77) | Ciprofloxacin | Single-dose | 0 | 0 | 0 | 0 | 0 | 0 |
| Singh et al. (2014) [33] | UK | 291 | Median 61 (Range 40-81) | Cefuroxime, Gentamicin, and Metronidazole | Single-dose | 3 | 0 | 0 | 0 | 0 | 3 |
| Simmons et al. (2019) [34] | UK | 236 | Mean 62.1 (Range 41.7-83.2) | NR | NR | 23 | NR | NR | NR | NR | 31  (8 skin infections) |
| Salagierski et al. (2019) [35] | Poland | 22 | Median 67 (Range 56-75) | Ciprofloxacin | 5 | 0 | 0 | 0 | 0 | 0 | 0 |
| Saito et al. (2017) [36] | Japan | Control: 449 Antiplatelet/ Anticoag: 149 | Mean 68.7 (SD 7.5) Mean 71.6 (SD 6.7) | Levofloxacin | 3 | 3 | 0 | 0 | 0 | 0 | 3 |
| Pepe et al. (2020) [37] | Italy | 875 | Median 63 (Range 47-78) | Cefazolin | Single-dose | 0 | 0 | 0 | 0 | 0 | 0 |
| Szabo et al. (2021) [38]* | US | 272 | Median 63 (Range 29–93) | Ceftriaxone or Ciprofloxacin (n=30, 12%) | Single-dose | 0 | 0 | 0 | 0 | 0 | 0 |
| Pepe et al. (2016) [39] | Italy | 1150 | Median 63 (Range 56-71) | Levofloxacin | 3 | 1 | 0 | 0 | 0 | 0 | 1 |
| Pepe et al. (2017) [40] | Italy | 150 | Median 62 (Range 47-78) | NR | NR | NR | NR | NR | NR | NR | NR |
| Muthuveloe et al. (2016) [41] | UK | 200 | Median 67 (Range 41-81) | Gentamicin and Metronidazole single-dose Ciprofloxacin x 3 days | 3 | NR | NR | NR | NR | NR | NR |
| Mischinger et al. (2018) [42] | Germany | 232 | Median 66 (IQR 60-73) | NR | NR | 0 | 0 | 0 | 0 | 0 | 0 |
| Miller et al. (2005) [100] | Australia | 81 | Mean 69.5 (95% CI +/- 1.4) | None |  | 0 | 0 | 0 | 0 | 0 | 0 |
| Miah et al. (2020) [43] | UK | 86 | Median 63.5, IQR 10.6 | Gentamicin | Single-dose | NR | NR | NR | NR | NR | NR |
| Meyer et al. (2018) [101] | US | 43 | Median 62 (Range 44-73) | None |  | 0 | 0 | 0 | 0 | 0 | 0 |
| Merrick et al. (2016) [44] | US | 46 | Median 63.5 | Ciprofloxacin | Single-dose | 0 | 0 | 0 | 0 | 0 | 0 |
| Merrick et al. (2017) [45] | US | 132 | Mean 63.8, Median 65.0 | NR | NR | NR | NR | NR | NR | NR | NR |
| Roberts et al. (2020) [46] | Australia | 774 | Mean 65.1 | Ciprofloxacin | Single-dose | 3 | 0 | 0 | 0 | 0 | 3 |
| Ristau et al. (2018) [47] | US | 1000 | Median 68 (IQR 61-74) | Cephalexin (60%) | Single-dose | 0 | NR | NR | NR | NR | 0 |
| Pepe et al. (2016) [48] | Italy | 75 | Median 66 (Range 58-73) | NR | NR | NR | NR | NR | NR | NR | NR |
| Pepe et al. (2014) [49] | Italy | 12 cores: 1428 18 cores: 1330 >24 cores: 630 Total: 4000 | Median 63.2 (Range 40-73) Median 61.8 (Range 49-74) Median 58.2 (Range 48-72) | Levofloxacin | 3 | 15  40  30  16 | NR | 3  8  7  4 | 3  9  10  6 | 2  8  8  5 | 151 |
| Pal et al. (2012) [50] | UK | 40 | Mean 62.9 (Range 49-73) | Co-amoxiclav and Gentamicin | Single-dose | 0 | 0 | 0 | 0 | 0 | 0 |
| Novella et al. (2003) [51] | Italy | TruGuide: 51 Conventional: 51 | Mean 63.7 (SD 7.14) Mean 65.9 (SD 7.54) | Trimethoprim/ Sulfamethoxazole or Fluoroquinolone | Single-dose | 0  3 | NR | NR | NR | 0  1 | 0 4 |
| Namekawa et al. (2015) [52] | Japan | 1663 | Median 68 (Range 30-86) | Sulbactam/ Cefoperazone | 3 | NR | NR | NR | NR | 28 | 28 |
| Nakai et al. (2017) [53] | Japan | 103 | Median 69 (Range 37-83) | Cefazolin | Single-dose | 0 | NR | NR | NR | NR | 0 |
| Martorana et al. (2015) [54] | Italy | 125 | Mean 64.73 (Range 52-81) | Ciprofloxacin | Single-dose | 0 | NR | NR | NR | 0 | 0 |
| Marra et al. (2020) [55] | Italy, China | 1327 | Mean 66.8 (SD 7.4) | Ciprofloxacin or Cefazolin | Single-dose | 1 | NR | NR | NR | 0 | 1 |
| Mai et al. (2016) [56] | China | 3007 | Mean 69.1 (Range 30-91) | Levofloxacin | 3 | NR | NR | NR | NR | NR | NR |
| Li et al. (2007) [57] | China | 303 | Mean 69.7 (Range 35-90) | NR | 3 | NR | NR | NR | NR | NR | NR |
| Kum et al. (2018) [58] | London | 176 | Mean 65 (Range 36-83) | Gentamicin | Single-dose | 0 | 0 | 0 | 0 | 0 | 0 |
| Losa et al. (2013) [59] | Italy | 87 | Median 63.9 (Range 46-78) | Cefoxitin | Single-dose | 0 | 0 | 0 | 0 | 0 | 0 |
| Lo et al. (2019) [60] | China | 100 | Mean 67.7 (SD 6.2) | Co-amoxiclav and Ciprofloxacin | 1 | NR | NR | NR | NR | NR | NR |
| Hadaschik et al. (2011) [61] | Germany | 106 | Median 66 (Range 42-83) | Fluoroquinolone | Single-dose | 0 | NR | NR | NR | 0 | 0 |
| Guo et al. (2015) [62] | China | 167 | Mean 67.2 (SD 6.8) | Ciprofloxacin | 2 | 0 | NR | NR | NR | 2 | 2 |
| Guo et al. (2017) [63] | China | 1300 | Mean 70.5 (Range 34-89) | Ciprofloxacin and Metronidazole | 1 | 5 | NR | NR | NR | 0 | 5 |
| Iremashvili et al. (2010) [64] | Russia | Local: 75 Local + nerve block: 75 | Mean 69.09 (SD 7.87) Mean 68.32 (SD 7.35) | Ciprofloxacin | 3 | NR | NR | NR | NR | 8 | 8 |
| Igel et al. (2001) [65] | USA | 88 | Mean 65 (Range 54-79) | Fluoroquinolone | 3 | 1 | NR | NR | NR | 3 | 4 |
| Huang et al. (2015) [66] | Australia | Active surveillance: 40 Prior negative: 54 Other: 17 | Mean 63.6 (Range 47-73) Mean 60.5 (Range 45-74) Mean 65 (Range 53-78) | Cefazolin | Single-dose | NR | NR | NR | NR | NR | NR |
| Huang et al. (2019) [102] | Taiwan | 130 | Mean 66.6 (SD 8.81) | None |  | 5 | NR | 0 | 1 | 0 | 6 |
| Hara et al. (2008) [67] | Japan | 126 | Mean 71 (SD 7.29) | Levofloxacin | Single-dose | NR | NR | NR | NR | 1 | 1 |
| Gorin et al. (2020) [103] | USA | 57 | Median 68.8 (Range 52-86.4) | Cefazolin (n = 1) | Single-dose | 0 | 0 | 0 | 0 | 0 | 0 |
| Gershman et al. (2013) [68] | USA | 34 | Mean: 66.2 (SD 6.4) | NR | NR | NR | NR | NR | NR | NR | NR |
| Furuno et al. (2004) [69] | Japan | 113 | Mean 64.5 (Range 50-81) | Fluoroquinolone | 5 | NR | NR | NR | NR | 0 | 0 |
| Emiliozzi et al. (2004) [70] | Italy | 6 cores: 107 12 cores: 107 | Median 67 Median 68 | Fluoroquinolone | 3 | NR | NR | NR | NR | NR | NR |
| Eldred et al. (2016) [71] | UK | 402 | Mean 61.1 (SD 8.7) | Aminoglycoside single-dose Fluoroquinolone x 3-5 days | 3-5 | NR | NR | NR | NR | NR | NR |
| Taira et al. (2010) [72] | USA | 373 | Median: 64.2 | NR | NR | NR | NR | NR | NR | NR | NR |
| Wetterauer et al. (2020) [73] | Switzerland | 400 | Median 66 (Range 49-86) | Fluoroquinolone the night before + 1 hour pre-op (29.5%) Fluoroquinoline 1 hour pre-op (26.2%) None (44.3%) | 0-1 | 0 | NR | NR | NR | NR | 0 |
| Ding et al. (2020) [104] | China | 2192 | Mean 67.63 (SD 7.11) | None |  | NR | NR | NR | NR | NR | 41 (unspecified) |
| DiBianco et al. (2016) [74] | USA | 244 | Mean 66.74 (SD 8.47) | Cefazolin | Single-dose | 0 | NR | NR | NR | 0 | 0 |
| Demura et al. (2005) [75] | Japan | 371 | Mean 67.4 (Range 43-87) | Fluoroquinolone | 5 | NR | NR | NR | NR | NR | NR |
| Danforth et al. (2012) [76] | USA | 137 | Mean 70 (Range 59-82) | Ciprofloxacin | 8 | NR | NR | NR | 1 | NR | 1 |
| Bittner et al. (2015) [77] | USA | 191 | Median 64.6 | Levofloxacin | 5 | NR | NR | 1 | NR | NR | 1 |
| Cronin et al. (2017) [78] | UK | 109 | Mean 65 (Range 46-84) | Co-amoxiclav | Single-dose | 0 | 0 | 0 | 0 | 0 | 0 |
| Bott et al. (2006) [79] | UK | 60 | Mean 64 (SD 6.4) | NR | NR | NR | NR | NR | NR | NR | NR |
| Bittner et al. (2013) [80] | USA | 485 | Median 65, Mean 64.8 | NR | NR | NR | NR | NR | NR | NR | NR |
| Merrick et al. (2020) [81] | USA | 226 | Mean 67.5 | NR | NR | NR | NR | NR | NR | NR | NR |
| Patel et al. (2020) [82] | Australia | 92 | Median 63 (IQR 58-68) | NR | NR | 0 | 0 | 0 | 0 | 0 | 0 |
| Tsivian et al. (2013) [83] | USA | 84 | Median 66.6 (IQR 59.4-69.5) | NR | Single-dose | 1 | NR | NR | 3 | NR | 4 |
| Pepdjonovic et al. (2017) [84] | Australia | 577 | Median 65 (Range 41-87) | Cephazolin | Single-dose | 1 | NR | NR | 1 | NR | 2 |
| Huang et al. (2016) [85] | China | 98 | Mean 63.4 (SD 9.81) | Cefuroxime | Single-dose | NR | NR | NR | NR | NR | 3 (unspecified) |
| Pinkstaff et al. (2005) [86] | USA | 210 | Mean 66.3 (Range 46-81) | Fluoroquinolone | 3 | NR | NR | NR | NR | NR | 0 |
| Chiu et al. (2020) [87] | China | 611 | Median 69 (IQR 65-72) | Co-amoxiclav and Ciprofloxacin | Single-dose | NR | NR | NR | NR | 2 | 2 |
| Cerruto et al. (2014) [88] | Italy | 54 | Mean 66.50 (SD 8.87) | Prulifloxacin | 3 | NR | NR | NR | NR | 0 | 0 |
| Taira et al. (2013) [89] | USA | 64 | Mean 68.1 (SD 13.5) | NR | NR | 0 | NR | NR | NR | NR | 0 |
| Bass et al. (2017) [90] | UK | 181 | Mean 68 (Range 46-92) | Gentamicin | Single-dose | NR | NR | NR | NR | NR | NR |
| Miah et al. (2018) [91] | UK | 249 | Mean 62 (SD 7) | Gentamicin and Cefuroxime | Single-dose | 23 | NR | NR | NR | NR | 23 |
| Babaei et al. (2016) [92] | Iran | 240 | Mean 64 (SD 8) | Fluoroquinolone | 4 | 0 | 0 | 0 | 0 | 0 | 0 |
| Baba et al. (2018) [93] | Japan | 485 | Median 70 (Range 41-90) | Cefazolin | 1 | NR | NR | 1 | 3 | NR | 4 |
| Asano et al. (2015) [94] | Japan | Local: 756 Spinal: 56 General: 3 | Median 69 (IQR 62-74) Median 72 (IQR 67-77) Median 70 (IQR 64-74) | Levofloxacin or Tosuflocazin | 1 | NR | NR | NR | 6 | NR | 6 |
| Jacewicz et al. (2020) [95] | Multinational | 377 | Mean 67 (95% CI 66-68) | Trimethoprim/ Sulfamethoxazole (39%) | NR | 1 | NR | NR | NR | NR | 1 |
| Bhatt et al. (2018) [96] | Ireland | 35 | Median 61 (Range 48-73) | Ciprofloxacin 12 hours preop Gentamicin periop | 1 | NR | NR | NR | NR | 4 | 4 |
| Sigle et al. (2021) [105] | Germany | 184 | Median 66.9 (IQR 61.8-72.0) | None |  | 2 | NR | NR | NR | 0 | 2 |
| Gunzel et al. (2021) [106] | Germany | 621 | Median 68 (IQR 62-74) | None |  | 3 | NR | NR | NR | NR | 3 |
| Lopez et al. (2021) [97] | UK, New Zealand, Hong Kong | 1043  175 | Median 68 (IQR 62-72) | Ciprofloxacin  None | Single-dose | NR | NR | NR | NR | NR | 0 |
| John et al. (2021) [98] | UK | 149  164 | Median 70 (IQR 66-74) | Ciprofloxacin  None | Single-dose | 0  0 | 0  0 | 0  0 | 0  0 | 0  0 | 0  0 |

IQR: interquartile range; SD: standard deviation; NR: not recorded; RCT: randomized controlled trial

* Studies with cohorts of men undergoing transperineal prostate biopsy with and without prophylactic antibiotics.

**References of Supplementary Tables**

[1] Pepe P, Cimino S, Garufi A, Priolo G, Russo GI, Giardina R, et al. Confirmatory biopsy of men under active surveillance: extended versus saturation versus multiparametric magnetic resonance imaging/transrectal ultrasound fusion prostate biopsy. Scand J Urol. 2017;51:260-3.

[2] Klatte T, Swietek N, Schatzl G, Waldert M. Transperineal template-guided biopsy for diagnosis of prostate cancer in patients with at least two prior negative biopsies. Wien Klin Wochenschr. 2013;125:669-73.

[3] Pepe P, Garufi A, Priolo GD, Galia A, Fraggetta F, Pennisi M. Is it Time to Perform Only Magnetic Resonance Imaging Targeted Cores? Our Experience with 1,032 Men Who Underwent Prostate Biopsy. J Urol. 2018;200:774-8.

[4] Emiliozzi P, Longhi S, Scarpone P, Pansadoro A, DePaula F, Pansadoro V. The value of a single biopsy with 12 transperineal cores for detecting prostate cancer in patients with elevated prostate specific antigen. J Urol. 2001;166:845-50.

[5] Pepe P, Pennisi M, Fraggetta F. Anterior prostate biopsy at initial and repeat evaluation: is it useful to detect significant prostate cancer? Int Braz J Urol. 2015;41:844-8.

[6] Yamamoto S, Kin U, Nakamura K, Hamano M, Nishikawa Y, Takenouchi T, et al. Transperineal ultrasound-guided 12-core systematic biopsy of the prostate for patients with a prostate-specific antigen level of 2.5-20 ng/ml in Japan. Int J Clin Oncol. 2005;10:117-21.

[7] Sivaraman A, Sanchez-Salas R, Ahmed HU, Barret E, Cathala N, Mombet A, et al. Clinical utility of transperineal template-guided mapping biopsy of the prostate after negative magnetic resonance imaging-guided transrectal biopsy. Urol Oncol. 2015;33:329 e7-11.

[8] Mehmood K, Mubarak M, Dhar M, Rafi M, Kinsella J. Transperineal template-guided prostate saturation biopsies in men with suspicion of prostate cancer: a pilot study from Pakistan. Malays J Pathol. 2017;39:285-8.

[9] Mabjeesh NJ, Lidawi G, Chen J, German L, Matzkin H. High detection rate of significant prostate tumours in anterior zones using transperineal ultrasound-guided template saturation biopsy. BJU Int. 2012;110:993-7.

[10] Kuru TH, Roethke MC, Seidenader J, Simpfendorfer T, Boxler S, Alammar K, et al. Critical evaluation of magnetic resonance imaging targeted, transrectal ultrasound guided transperineal fusion biopsy for detection of prostate cancer. J Urol. 2013;190:1380-6.

[11] Kum F, Jones A, Nigam R. Factors influencing urinary retention after transperineal template biopsy of the prostate: outcomes from a regional cancer centre. World J Urol. 2019;37:337-42.

[12] Garcia Bennett J, Vilanova JC, Guma Padro J, Parada D, Conejero A. Evaluation of MR imaging-targeted biopsies of the prostate in biopsy-naive patients. A single centre study. Diagn Interv Imaging. 2017;98:677-84.

[13] Ekwueme K, Simpson H, Zakhour H, Parr NJ. Transperineal template-guided saturation biopsy using a modified technique: outcome of 270 cases requiring repeat prostate biopsy. BJU Int. 2013;111:E365-73.

[14] Bigliocchi M, Marini M, Nofroni I, Perugia G, Shahabadi H, Ciccariello M. Prostate cancer detection rate of transrectal ultrasonography, digital rectal examination, and prostate-specific antigen: results of a five-year study of 6- versus 12-core transperineal prostate biopsy. Minerva Urol Nefrol. 2007;59:395-402; 3-6.

[15] Zhang F, Shao Q, Du Y, Tian Y. Evaluation of 24-core coaxial needle saturation biopsy of the prostate by the transperineal approach in detecting prostate cancer in patients without previous biopsy history: A single-center report. J Cancer Res Ther. 2019;15:380-5.

[16] Young R, Norris B, Reeves F, Peters JS. A Retrospective Comparison of Transrectal and Transperineal Prostate Biopsies: Experience of a Single Surgeon. J Endourol. 2019;33:498-502.

[17] Yazici S, Kiziloz H, Bozaci AC, Baydar DE, Del Biondo D, Ozen H. Predictors of prostate cancer in ultrasound-guided transperineal saturation biopsy in Turkish men with multiple prior negative biopsies. Urologia. 2016;83:71-6.

[18] Yang X, Lee AY, Law YM, Sim ASP, Tay KJ, Lau WKO, et al. Stereotactic robot-assisted transperineal prostate biopsy under local anaesthesia and sedation: moving robotic biopsy from operating theatre to clinic. J Robot Surg. 2020;14:767-72.

[19] Wang L, Wang X, Zhao W, Zhao Z, Li Z, Fei S, et al. Surface-projection-based transperineal cognitive fusion targeted biopsy of the prostate: an original technique with a good cancer detection rate. BMC Urol. 2019;19:107.

[20] Wajswol E, Winoker JS, Anastos H, Falagario U, Okhawere K, Martini A, et al. A cohort of transperineal electromagnetically tracked magnetic resonance imaging/ultrasonography fusion-guided biopsy: assessing the impact of inter-reader variability on cancer detection. BJU Int. 2020;125:531-40.

[21] Wadhwa K, Carmona-Echeveria L, Kuru T, Gaziev G, Serrao E, Parashar D, et al. Transperineal prostate biopsies for diagnosis of prostate cancer are well tolerated: a prospective study using patient-reported outcome measures. Asian J Androl. 2017;19:62-6.

[22] Vyas L, Acher P, Kinsella J, Challacombe B, Chang RT, Sturch P, et al. Indications, results and safety profile of transperineal sector biopsies (TPSB) of the prostate: a single centre experience of 634 cases. BJU Int. 2014;114:32-7.

[23] Voss J, Pal R, Ahmed S, Hannah M, Jaulim A, Walton T. Utility of early transperineal template-guided prostate biopsy for risk stratification in men undergoing active surveillance for prostate cancer. BJU Int. 2018;121:863-70.

[24] Takenaka A, Hara R, Ishimura T, Fujii T, Jo Y, Nagai A, et al. A prospective randomized comparison of diagnostic efficacy between transperineal and transrectal 12-core prostate biopsy. Prostate Cancer Prostatic Dis. 2008;11:134-8.

[25] Wegelin O, Exterkate L, van der Leest M, Kelder JC, Bosch J, Barentsz JO, et al. Complications and Adverse Events of Three Magnetic Resonance Imaging-based Target Biopsy Techniques in the Diagnosis of Prostate Cancer Among Men with Prior Negative Biopsies: Results from the FUTURE Trial, a Multicentre Randomised Controlled Trial. Eur Urol Oncol. 2019;2:617-24.

[26] Symons JL, Huo A, Yuen CL, Haynes AM, Matthews J, Sutherland RL, et al. Outcomes of transperineal template-guided prostate biopsy in 409 patients. BJU Int. 2013;112:585-93.

[27] Suzuki M, Kawakami S, Asano T, Masuda H, Saito K, Koga F, et al. Safety of transperineal 14-core systematic prostate biopsy in diabetic men. Int J Urol. 2009;16:930-5.

[28] Stefanova V, Buckley R, Flax S, Spevack L, Hajek D, Tunis A, et al. Transperineal Prostate Biopsies Using Local Anesthesia: Experience with 1,287 Patients. Prostate Cancer Detection Rate, Complications and Patient Tolerability. J Urol. 2019;201:1121-6.

[29] Song W, Kang M, Jeong BC, Seo SI, Jeon SS, Lee HM, et al. The clinical utility of transperineal template-guided saturation prostate biopsy for risk stratification after transrectal ultrasound-guided biopsy. Investig Clin Urol. 2019;60:454-62.

[30] Smith JB, Popert R, Nuttall MC, Vyas L, Kinsella J, Cahill D. Transperineal sector prostate biopsies: a local anesthetic outpatient technique. Urology. 2014;83:1344-9.

[31] Togo Y, Kubo T, Taoka R, Hiyama Y, Uehara T, Hashimoto J, et al. Occurrence of infection following prostate biopsy procedures in Japan: Japanese Research Group for Urinary Tract Infection (JRGU) - a multi-center retrospective study. J Infect Chemother. 2014;20:232-7.

[32] Thurtle D, Starling L, Leonard K, Stone T, Gnanapragasam VJ. Improving the safety and tolerability of local anaesthetic outpatient transperineal prostate biopsies: A pilot study of the CAMbridge PROstate Biopsy (CAMPROBE) method. J Clin Urol. 2018;11:192-9.

[33] Singh PB, Anele C, Dalton E, Barbouti O, Stevens D, Gurung P, et al. Prostate cancer tumour features on template prostate-mapping biopsies: implications for focal therapy. Eur Urol. 2014;66:12-9.

[34] Simmons LAM, Kanthabalan A, Arya M, Briggs T, Charman SC, Freeman A, et al. Prostate Imaging Compared to Transperineal Ultrasound-guided biopsy for significant prostate cancer Risk Evaluation (PICTURE): a prospective cohort validating study assessing Prostate HistoScanning. Prostate Cancer Prostatic Dis. 2019;22:261-7.

[35] Salagierski M, Kania P, Wierzcholowski W, Pozniak-Balicka R. The role of a template-assisted cognitive transperineal prostate biopsy technique in patients with benign transrectal prostate biopsies: a preliminary experience. Cent European J Urol. 2019;72:15-8.

[36] Saito K, Washino S, Nakamura Y, Konishi T, Ohshima M, Arai Y, et al. Transperineal ultrasound-guided prostate biopsy is safe even when patients are on combination antiplatelet and/or anticoagulation therapy. BMC Urol. 2017;17:53.

[37] Pepe P, Pennisi M, Fraggetta F. How Many Cores Should be Obtained During Saturation Biopsy in the Era of Multiparametric Magnetic Resonance? Experience in 875 Patients Submitted to Repeat Prostate Biopsy. Urology. 2020;137:133-7.

[38] Szabo RJ. Free-Hand Transperineal Prostate Biopsy Under Local Anesthesia in the Office Without Antibiotic Prophylaxis: Experience with 304 Cases. J Endourol. 2021;35:518-24.

[39] Pepe P, Pennisi M. Erectile dysfunction in 1050 men following extended (18 cores) vs saturation (28 cores) vs saturation plus MRI-targeted prostate biopsy (32 cores). Int J Impot Res. 2016;28:1-3.

[40] Pepe P, Garufi A, Priolo GD, Pennisi M. Multiparametric MRI/TRUS Fusion Prostate Biopsy: Advantages of a Transperineal Approach. Anticancer Res. 2017;37:3291-4.

[41] Muthuveloe D, Telford R, Viney R, Patel P. The detection and upgrade rates of prostate adenocarcinoma following transperineal template-guided prostate biopsy - a tertiary referral centre experience. Cent European J Urol. 2016;69:42-7.

[42] Mischinger J, Kaufmann S, Russo GI, Harland N, Rausch S, Amend B, et al. Targeted vs systematic robot-assisted transperineal magnetic resonance imaging-transrectal ultrasonography fusion prostate biopsy. BJU Int. 2018;121:791-8.

[43] Miah S, Servian P, Patel A, Lovegrove C, Skelton L, Shah TT, et al. A prospective analysis of robotic targeted MRI-US fusion prostate biopsy using the centroid targeting approach. J Robot Surg. 2020;14:69-74.

[44] Merrick GS, Irvin S, Fiano R, Anderson R, Butler WM, Adamovich E. Pathology and Quality of Life Outcomes Following Office-based Transperineal Prostate Biopsy. Urology. 2016;94:24-8.

[45] Merrick GS, Galbreath RW, Bennett A, Butler WM, Amamovich E. Incidence, grade and distribution of prostate cancer following transperineal template-guided mapping biopsy in patients with atypical small acinar proliferation. World J Urol. 2017;35:1009-13.

[46] Roberts MJ, Macdonald A, Ranasinghe S, Bennett H, Teloken PE, Harris P, et al. Transrectal versus transperineal prostate biopsy under intravenous anaesthesia: a clinical, microbiological and cost analysis of 2048 cases over 11 years at a tertiary institution. Prostate Cancer Prostatic Dis. 2021;24:169-76.

[47] Ristau BT, Allaway M, Cendo D, Hart J, Riley J, Parousis V, et al. Free-hand transperineal prostate biopsy provides acceptable cancer detection and minimizes risk of infection: evolving experience with a 10-sector template. Urol Oncol. 2018;36:528 e15- e20.

[48] Pepe P, Cimino S, Garufi A, Priolo G, Russo GI, Giardina R, et al. Detection rate for significant cancer at confirmatory biopsy in men enrolled in Active Surveillance protocol: 20 cores vs 30 cores vs MRI/TRUS fusion prostate biopsy. Arch Ital Urol Androl. 2016;88:300-3.

[49] Pepe P, Aragona F. Prostate biopsy: results and advantages of the transperineal approach--twenty-year experience of a single center. World J Urol. 2014;32:373-7.

[50] Pal RP, Elmussareh M, Chanawani M, Khan MA. The role of a standardized 36 core template-assisted transperineal prostate biopsy technique in patients with previously negative transrectal ultrasonography-guided prostate biopsies. BJU Int. 2012;109:367-71.

[51] Novella G, Ficarra V, Galfano A, Ballario R, Novara G, Cavalleri S, et al. Pain assessment after original transperineal prostate biopsy using a coaxial needle. Urology. 2003;62:689-92.

[52] Namekawa T, Fukasawa S, Komaru A, Kobayashi M, Imamura Y, Ohzeki T, et al. Prospective evaluation of the safety of transrectal ultrasound-guided transperineal prostate biopsy based on adverse events. Int J Clin Oncol. 2015;20:1185-91.

[53] Nakai Y, Tanaka N, Anai S, Miyake M, Hori S, Tatsumi Y, et al. Transperineal template-guided saturation biopsy aimed at sampling one core for each milliliter of prostate volume: 103 cases requiring repeat prostate biopsy. BMC Urol. 2017;17:28.

[54] Martorana E, Micali S, Ghaith A, Reggiani Bonetti L, Sighinolfi MC, Galli R, et al. Advantages of single-puncture transperineal saturation biopsy of prostate: analysis of outcomes in 125 patients using our scheme. Int Urol Nephrol. 2015;47:735-41.

[55] Marra G, Zhuang J, Beltrami M, Calleris G, Zhao X, Marquis A, et al. Transperineal freehand multiparametric MRI fusion targeted biopsies under local anaesthesia for prostate cancer diagnosis: a multicentre prospective study of 1014 cases. BJU Int. 2021;127:122-30.

[56] Mai Z, Yan W, Zhou Y, Zhou Z, Chen J, Xiao Y, et al. Transperineal template-guided prostate biopsy: 10 years of experience. BJU Int. 2016;117:424-9.

[57] Li H, Yan W, Zhou Y, Ji Z, Chen J. Transperineal ultrasound-guided saturation biopsies using 11-region template of prostate: report of 303 cases. Urology. 2007;70:1157-61.

[58] Kum F, Elhage O, Maliyil J, Wong K, Faure Walker N, Kulkarni M, et al. Initial outcomes of local anaesthetic freehand transperineal prostate biopsies in the outpatient setting. BJU Int. 2020;125:244-52.

[59] Losa A, Gadda GM, Lazzeri M, Lughezzani G, Cardone G, Freschi M, et al. Complications and quality of life after template-assisted transperineal prostate biopsy in patients eligible for focal therapy. Urology. 2013;81:1291-6.

[60] Lo KL, Chui KL, Leung CH, Ma SF, Lim K, Ng T, et al. Outcomes of transperineal and transrectal ultrasound-guided prostate biopsy. Hong Kong Med J. 2019;25:209-15.

[61] Hadaschik BA, Kuru TH, Tulea C, Rieker P, Popeneciu IV, Simpfendorfer T, et al. A novel stereotactic prostate biopsy system integrating pre-interventional magnetic resonance imaging and live ultrasound fusion. J Urol. 2011;186:2214-20.

[62] Guo LH, Wu R, Xu HX, Xu JM, Wu J, Wang S, et al. Comparison between Ultrasound Guided Transperineal and Transrectal Prostate Biopsy: A Prospective, Randomized, and Controlled Trial. Sci Rep. 2015;5:16089.

[63] Guo G, Xu Y, Zhang X. TRUS-guided transperineal prostate 12+X core biopsy with template for the diagnosis of prostate cancer. Oncol Lett. 2017;13:4863-7.

[64] Iremashvili VV, Chepurov AK, Kobaladze KM, Gamidov SI. Periprostatic local anesthesia with pudendal block for transperineal ultrasound-guided prostate biopsy: a randomized trial. Urology. 2010;75:1023-7.

[65] Igel TC, Knight MK, Young PR, Wehle MJ, Petrou SP, Broderick GA, et al. Systematic transperineal ultrasound guided template biopsy of the prostate in patients at high risk. J Urol. 2001;165:1575-9.

[66] Huang S, Reeves F, Preece J, Satasivam P, Royce P, Grummet JP. Significant impact of transperineal template biopsy of the prostate at a single tertiary institution. Urol Ann. 2015;7:428-32.

[67] Hara R, Jo Y, Fujii T, Kondo N, Yokoyoma T, Miyaji Y, et al. Optimal approach for prostate cancer detection as initial biopsy: prospective randomized study comparing transperineal versus transrectal systematic 12-core biopsy. Urology. 2008;71:191-5.

[68] Gershman B, Zietman AL, Feldman AS, McDougal WS. Transperineal template-guided prostate biopsy for patients with persistently elevated PSA and multiple prior negative biopsies. Urol Oncol. 2013;31:1093-7.

[69] Furuno T, Demura T, Kaneta T, Gotoda H, Muraoka S, Sato T, et al. Difference of cancer core distribution between first and repeat biopsy: In patients diagnosed by extensive transperineal ultrasound guided template prostate biopsy. Prostate. 2004;58:76-81.

[70] Emiliozzi P, Scarpone P, DePaula F, Pizzo M, Federico G, Pansadoro A, et al. The incidence of prostate cancer in men with prostate specific antigen greater than 4.0 ng/ml: a randomized study of 6 versus 12 core transperineal prostate biopsy. J Urol. 2004;171:197-9.

[71] Eldred-Evans D, Kasivisvanathan V, Khan F, Hemelrijck MV, Polson A, Acher P, et al. The Use of Transperineal Sector Biopsy as A First-Line Biopsy Strategy: A Multi-Institutional Analysis of Clinical Outcomes and Complications. Urol J. 2016;13:2849-55.

[72] Taira AV, Merrick GS, Galbreath RW, Andreini H, Taubenslag W, Curtis R, et al. Performance of transperineal template-guided mapping biopsy in detecting prostate cancer in the initial and repeat biopsy setting. Prostate Cancer Prostatic Dis. 2010;13:71-7.

[73] Wetterauer C, Shahin O, Federer-Gsponer JR, Keller N, Wyler S, Seifert HH, et al. Feasibility of freehand MRI/US cognitive fusion transperineal biopsy of the prostate in local anaesthesia as in-office procedure-experience with 400 patients. Prostate Cancer Prostatic Dis. 2020;23:429-34.

[74] DiBianco JM, Mullins JK, Allaway M. Ultrasound Guided, Freehand Transperineal Prostate Biopsy: An Alternative to the Transrectal Approach. Urology Practice. 2016;3:134-40.

[75] Demura T, Hioka T, Furuno T, Kaneta T, Gotoda H, Muraoka S, et al. Differences in tumor core distribution between palpable and nonpalpable prostate tumors in patients diagnosed using extensive transperineal ultrasound-guided template prostate biopsy. Cancer. 2005;103:1826-32.

[76] Danforth TL, Chevli KK, Baumann L, Duff M. Low incidence of prostate cancer identified in the transition and anterior zones with transperineal biopsy. Res Rep Urol. 2012;4:71-6.

[77] Bittner N, Merrick GS, Bennett A, Butler WM, Andreini HJ, Taubenslag W, et al. Diagnostic Performance of Initial Transperineal Template-guided Mapping Biopsy of the Prostate Gland. Am J Clin Oncol. 2015;38:300-3.

[78] Cronin T, Neill L, Nelson J, Stewart R, Sangster P, Khoubehi B. Complications of transperineal template-guided prostate biopsy: A single centre experience in 109 cases. Surgical Practice. 2017;21:103-6.

[79] Bott SR, Henderson A, Halls JE, Montgomery BS, Laing R, Langley SE. Extensive transperineal template biopsies of prostate: modified technique and results. Urology. 2006;68:1037-41.

[80] Bittner N, Merrick GS, Butler WM, Bennett A, Galbreath RW. Incidence and pathological features of prostate cancer detected on transperineal template guided mapping biopsy after negative transrectal ultrasound guided biopsy. J Urol. 2013;190:509-14.

[81] Merrick GS, Tennant A, Fiano R, Bennett A, Anderson R, Galbreath R, et al. Active surveillance outcomes in prostate cancer patients: the use of transperineal template-guided mapping biopsy for patient selection. World J Urol. 2020;38:361-9.

[82] Patel MI, Muter S, Vladica P, Gillatt D. Robotic-assisted magnetic resonance imaging ultrasound fusion results in higher significant cancer detection compared to cognitive prostate targeting in biopsy naive men. Transl Androl Urol. 2020;9:601-8.

[83] Tsivian M, Abern MR, Qi P, Polascik TJ. Short-term functional outcomes and complications associated with transperineal template prostate mapping biopsy. Urology. 2013;82:166-70.

[84] Pepdjonovic L, Tan GH, Huang S, Mann S, Frydenberg M, Moon D, et al. Zero hospital admissions for infection after 577 transperineal prostate biopsies using single-dose cephazolin prophylaxis. World J Urol. 2017;35:1199-203.

[85] Huang H, Wang W, Lin T, Zhang Q, Zhao X, Lian H, et al. Comparison of the complications of traditional 12 cores transrectal prostate biopsy with image fusion guided transperineal prostate biopsy. BMC Urol. 2016;16:68.

[86] Pinkstaff DM, Igel TC, Petrou SP, Broderick GA, Wehle MJ, Young PR. Systematic transperineal ultrasound-guided template biopsy of the prostate: three-year experience. Urology. 2005;65:735-9.

[87] Chiu PK, Lo KL, Teoh JY, Ma SF, Leung CH, Wong HF, et al. Sectoral cancer detection and tolerability of freehand transperineal prostate biopsy under local anaesthesia. Prostate Cancer Prostatic Dis. 2021;24:431-8.

[88] Cerruto MA, Vianello F, D'Elia C, Artibani W, Novella G. Transrectal versus transperineal 14-core prostate biopsy in detection of prostate cancer: a comparative evaluation at the same institution. Arch Ital Urol Androl. 2014;86:284-7.

[89] Taira AV, Merrick GS, Bennett A, Andreini H, Taubenslag W, Galbreath RW, et al. Transperineal template-guided mapping biopsy as a staging procedure to select patients best suited for active surveillance. Am J Clin Oncol. 2013;36:116-20.

[90] Bass EJ, Donaldson IA, Freeman A, Jameson C, Punwani S, Moore C, et al. Magnetic resonance imaging targeted transperineal prostate biopsy: a local anaesthetic approach. Prostate Cancer Prostatic Dis. 2017;20:311-7.

[91] Miah S, Eldred-Evans D, Simmons LAM, Shah TT, Kanthabalan A, Arya M, et al. Patient Reported Outcome Measures for Transperineal Template Prostate Mapping Biopsies in the PICTURE Study. J Urol. 2018;200:1235-40.

[92] Babaei Jandaghi A, Habibzadeh H, Falahatkar S, Heidarzadeh A, Pourghorban R. Transperineal Prostate Core Needle Biopsy: A Comparison of Coaxial Versus Noncoaxial Method in a Randomised Trial. Cardiovasc Intervent Radiol. 2016;39:1736-42.

[93] Baba K, Sekine Y, Miyazawa Y, Syuto T, Nomura M, Koike H, et al. Assessment of antimicrobiral prophylaxis in transperineal prostate biopsy: A single-center retrospective study of 485 cases. J Infect Chemother. 2018;24:637-40.

[94] Asano T, Kobayashi S, Yano M, Otsuka Y, Kitahara S. Continued administration of antithrombotic agents during transperineal prostate biopsy. Int Braz J Urol. 2015;41:116-23.

[95] Jacewicz M, Gunzel K, Rud E, Lauritzen PM, Galtung KF, Hinz S, et al. Multicenter transperineal MRI-TRUS fusion guided outpatient clinic prostate biopsies under local anesthesia. Urol Oncol. 2021;39:432 e1- e7.

[96] Bhatt NR, Breen K, Haroon UM, Akram M, Flood HD, Giri SK. Patient experience after transperineal template prostate biopsy compared to prior transrectal ultrasound guided prostate biopsy. Cent European J Urol. 2018;71:43-7.

[97] Lopez JF, Campbell A, Omer A, Stroman L, Bondad J, Austin T, et al. Local anaesthetic transperineal (LATP) prostate biopsy using a probe-mounted transperineal access system: a multicentre prospective outcome analysis. BJU Int. 2021;128:311-8.

[98] John JB, MacCormick A, MacDonagh R, Speakman MJ, Vennam R, Burns-Cox N. Complications following local anaesthetic transperineal prostate biopsies without antibiotic prophylaxis: An institution’s experience. Journal of Clinical Urology.0:2051415820987661.

[99] Dimmen M, Vlatkovic L, Hole KH, Nesland JM, Brennhovd B, Axcrona K. Transperineal prostate biopsy detects significant cancer in patients with elevated prostate-specific antigen (PSA) levels and previous negative transrectal biopsies. BJU Int. 2012;110:E69-75.

[100] Miller J, Perumalla C, Heap G. Complications of transrectal versus transperineal prostate biopsy. ANZ J Surg. 2005;75:48-50.

[101] Meyer AR, Joice GA, Schwen ZR, Partin AW, Allaf ME, Gorin MA. Initial Experience Performing In-office Ultrasound-guided Transperineal Prostate Biopsy Under Local Anesthesia Using the PrecisionPoint Transperineal Access System. Urology. 2018;115:8-13.

[102] Huang GL, Kang CH, Lee WC, Chiang PH. Comparisons of cancer detection rate and complications between transrectal and transperineal prostate biopsy approaches - a single center preliminary study. BMC Urol. 2019;19:101.

[103] Gorin MA, Meyer AR, Zimmerman M, Harb R, Joice GA, Schwen ZR, et al. Transperineal prostate biopsy with cognitive magnetic resonance imaging/biplanar ultrasound fusion: description of technique and early results. World J Urol. 2020;38:1943-9.

[104] Ding XF, Luan Y, Lu SM, Zhou GC, Huang TB, Zhu LY, et al. Risk factors for infection complications after transrectal ultrasound-guided transperineal prostate biopsy. World J Urol. 2021;39:2463-7.

[105] Sigle A, Suarez-Ibarrola R, Pudimat M, Michaelis J, Jilg CA, Miernik A, et al. Safety and side effects of transperineal prostate biopsy without antibiotic prophylaxis. Urol Oncol. 2021.

[106] Gunzel K, Magheli A, Baco E, Cash H, Heinrich S, Neubert H, et al. Infection rate and complications after 621 transperineal MRI-TRUS fusion biopsies in local anesthesia without standard antibiotic prophylaxis. World J Urol. 2021.
