# Supplementary material for: Role of Prophylactic Antibiotics in Transperineal Prostate Biopsy: A Systematic Review and Meta-analysis
Source: Eur Urol Open Sci. 2022 Jan 29;37:53–63. doi: 10.1016/j.euros.2022.01.001 (PMC8883190; doi:10.1016/j.euros.2022.01.001)
Supplement: Supplementary data 4 [file mmc4.docx]

**MEDLINE Search Strategy**

1. "transperineal"[All Fields] OR "transperineally"[All Fields] OR "perinal"[Supplementary Concept] OR "perinal"[All Fields] OR "perineally"[All Fields] OR "Perineum"[MeSH Terms] OR "Perineum"[All Fields] OR "perineal"[All Fields] OR "Perineum"[MeSH Terms] OR "Perineum"[All Fields] OR "perineums"[All Fields] OR "Perineum"[MeSH Terms]
2. "biopsie"[All Fields] OR "Biopsy"[MeSH Terms] OR "Biopsy"[All Fields] OR "biopsied"[All Fields] OR "biopsies"[All Fields] OR "biopsy s"[All Fields] OR "biopsying"[All Fields] OR "biopsys"[All Fields] OR "pathology"[MeSH Subheading] OR "pathology"[All Fields] OR "biopsie"[All Fields] OR "Biopsy"[MeSH Terms] OR "Biopsy"[All Fields] OR "biopsied"[All Fields] OR "biopsies"[All Fields] OR "biopsy s"[All Fields] OR "biopsying"[All Fields] OR "biopsys"[All Fields] OR "pathology"[MeSH Subheading] OR "pathology"[All Fields] OR "Biopsy"[MeSH Terms:noexp] OR "biopsy, needle"[MeSH Terms] OR "biopsy, large core needle"[MeSH Terms] OR "biopsy, fine needle"[MeSH Terms]
3. "prostat"[All Fields] OR "Prostate"[MeSH Terms] OR "Prostate"[All Fields] OR "prostates"[All Fields] OR "prostatic"[All Fields] OR "prostatism"[MeSH Terms] OR "prostatism"[All Fields] OR "prostatitis"[MeSH Terms] OR "prostatitis"[All Fields] OR "prostate cancer"[All Fields] OR "prostate cancers"[All Fields] OR "prostate neoplasm"[All Fields] OR "prostate neoplasms"[All Fields] OR "prostate tumor"[All Fields] OR "prostate tumors"[All Fields] OR "prostate tumour"[All Fields] OR "prostate tumours"[All Fields] OR "Prostate"[MeSH Terms] OR "Prostatic Neoplasms"[MeSH Terms]
4. "infection*"[All Fields] OR "infect"[All Fields] OR "infectability"[All Fields] OR "infectable"[All Fields] OR "infectant"[All Fields] OR "infectants"[All Fields] OR "infected"[All Fields] OR "infecteds"[All Fields] OR "infectibility"[All Fields] OR "infectible"[All Fields] OR "infecting"[All Fields] OR "infection s"[All Fields] OR "Infections"[MeSH Terms] OR "Infections"[All Fields] OR "infection"[All Fields] OR "infective"[All Fields] OR "infectiveness"[All Fields] OR "infectives"[All Fields] OR "infectivities"[All Fields] OR "infects"[All Fields] OR "pathogenicity"[MeSH Subheading] OR "pathogenicity"[All Fields] OR "infectivity"[All Fields] OR "infectious"[All Fields] OR "infectiousness"[All Fields] OR "Fever"[MeSH Terms] OR "Fever"[All Fields] OR "fevers"[All Fields] OR "Sepsis"[MeSH Terms] OR "Sepsis"[All Fields] OR "urinary tract infection"[All Fields] OR "Urinary Tract Infections"[All Fields] OR "Abscess"[MeSH Terms] OR "Abscess"[All Fields] OR "abscesses"[All Fields] OR "abscessation"[All Fields] OR "abscessed"[All Fields] OR "abscessing"[All Fields] OR "prostat"[All Fields] OR "prostate"[MeSH Terms] OR "prostate"[All Fields] OR "prostates"[All Fields] OR "prostatic"[All Fields] OR "prostatism"[MeSH Terms] OR "prostatism"[All Fields] OR "Prostatitis"[MeSH Terms] OR "Prostatitis"[All Fields] OR "Orchitis"[MeSH Terms] OR "Orchitis"[All Fields] OR "epididymes"[All Fields] OR "epididymis"[MeSH Terms] OR "epididymis"[All Fields] OR "epididymal"[All Fields] OR "Epididymitis"[MeSH Terms] OR "Epididymitis"[All Fields] OR "epididymitides"[All Fields] OR "Epididymo-orchitis"[All Fields] OR "Epididymoorchitis"[All Fields] OR "Pyelonephritis"[MeSH Terms] OR "Pyelonephritis"[All Fields] OR "pyelonephritides"[All Fields] OR "complicances"[All Fields] OR "complicate"[All Fields] OR "complicated"[All Fields] OR "complicates"[All Fields] OR "complicating"[All Fields] OR "complication"[All Fields] OR "complication s"[All Fields] OR "complications"[MeSH Subheading] OR "complications"[All Fields] OR "complicances"[All Fields] OR "complicate"[All Fields] OR "complicated"[All Fields] OR "complicates"[All Fields] OR "complicating"[All Fields] OR "complication"[All Fields] OR "complication s"[All Fields] OR "complications"[MeSH Subheading] OR "complications"[All Fields] OR "Infections"[MeSH Terms] OR "Surgical Wound Infection"[MeSH Terms] OR "Fever"[MeSH Terms] OR "Sepsis"[MeSH Terms] OR "Urinary Tract Infections"[MeSH Terms] OR "Abscess"[MeSH Terms:noexp] OR "Prostatitis"[MeSH Terms] OR "Orchitis"[MeSH Terms] OR "Epididymitis"[MeSH Terms] OR "Pyelonephritis"[MeSH Terms]
5. 1 AND 2 AND 3 AND 4
